# The direct and indirect effects of bioactive compounds against coronavirus

**DOI:** 10.1002/fft2.119

**Published:** 2021-12-08

**Authors:** Merve Tomas, Esra Capanoglu, Akbar Bahrami, Hamed Hosseini, Safoura Akbari‐Alavijeh, Rezvan Shaddel, Abdur Rehman, Atefe Rezaei, Ali Rashidinejad, Farhad Garavand, Mostafa Goudarzi, Seid Mahdi Jafari

**Affiliations:** ^1^ Department of Food Engineering Faculty of Engineering and Natural Sciences Istanbul Sabahattin Zaim University Halkali Istanbul Turkey; ^2^ Department of Food Engineering Faculty of Chemical and Metallurgical Engineering Istanbul Technical University Maslak Istanbul Turkey; ^3^ Center for Excellence in Post‐Harvest Technologies North Carolina Agricultural and Technical State University Kannapolis North Carolina USA; ^4^ Food Additives Department Food Science and Technology Research Institute Research Center for Iranian Academic Center for Education Culture and Research (ACECR) Mashhad Iran; ^5^ Department of Food Science and Technology Faculty of Agriculture and Natural Resources University of Mohaghegh Ardabili Ardabil Iran; ^6^ State Key Laboratory of Food Science and Technology Jiangnan University Jiangsu Wuxi China; ^7^ Collaborative Innovation Centre of Food Safety and Quality Control Wuxi Jiangsu Province China; ^8^ Department of Food Science and Technology School of Nutrition and Food Science Isfahan University of Medical Sciences Isfahan Iran; ^9^ Riddet Institute Massey University Palmerston North New Zealand; ^10^ Department of Food Chemistry and Technology Teagasc Food Research Centre, Moorepark Fermoy, Co. Cork Ireland; ^11^ Department of Food Science and Engineering University College of Agriculture and Natural Resources University of Tehran Karaj Iran; ^12^ Department of Food Materials and Process Design Engineering Gorgan University of Agricultural Science and Natural Resources Gorgan Iran

**Keywords:** antiviral activity, bioactive compounds, coronavirus, COVID‐19, functional foods, immune system

## Abstract

Emerging viruses are known to pose a threat to humans in the world. COVID‐19, a newly emerging viral respiratory disease, can spread quickly from people to people via respiratory droplets, cough, sneeze, or exhale. Up to now, there are no specific therapies found for the treatment of COVID‐19. In this sense, the rising demand for effective antiviral drugs is stressed. The main goal of the present study is to cover the current literature about bioactive compounds (e.g., polyphenols, glucosinolates, carotenoids, minerals, vitamins, oligosaccharides, bioactive peptides, essential oils, and probiotics) with potential efficiency against COVID‐19, showing antiviral activities via the inhibition of coronavirus entry into the host cell, coronavirus enzymes, as well as the virus replication in human cells. In turn, these compounds can boost the immune system, helping fight against COVID‐19. Overall, it can be concluded that bioactives and the functional foods containing these compounds can be natural alternatives for boosting the immune system and defeating coronavirus.

## Graphical abstract

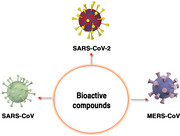

## 1 INTRODUCTION

Epidemiologically, natural bioactive compounds provide protection and decrease the risk of various chronic diseases such as cardiovascular disease, cancer, diabetes, and obesity (Gonzalez, 2020). On the other hand, viral infections are the most fatal forms of diseases and some of their forms still cannot be completely treated (e.g., hepatitis and human immunodeficiency virus (HIV)). At present, the novel coronavirus severe acute respiratory syndrome coronavirus 2 (SARS‐CoV‐2), a newly emerging viral respiratory disease, is known to cause COVID‐19, which is terribly spreading around the globe and there is no stoppage (Duda‐Chodak et al., 2020). SARS‐CoV‐2 is an enveloped virus with a positive‐sense, single‐stranded RNA genome of ∼30 kb. SARS‐CoV‐2 belongs to the genus betacoronavirus, together with SARS‐CoV and Middle East respiratory syndrome coronavirus (MERS‐CoV) (Jo et al., 2020). Person‐to‐person spread of SARS‐CoV, MERS‐CoV, and SARS‐CoV‐2 mainly occurs via respiratory droplets produced when an infected person coughs or sneezes (Yan et al., 2020). By September 12, 2021, about 239 million COVID‐19 infection cases and more than 4.8 million associated deaths have been reported in the world (University, 2020). The disease is easily transmitted from person to person via respiratory droplets, cough, sneeze, or exhale, and the incubation period ranges from 2 to 14 days. The symptoms of COVID‐19, which appear approximately five days after infection, are usually cough, loss of taste or smell, high fever, fatigue, breathlessness, and others (Singhal, 2020). However, no specific vaccine or therapy has yet been approved for humans against COVID‐19. Virus replication takes place within the cell, and to enter the cell, the virus first attaches to the host cellular receptor angiotensin‐converting enzyme 2 (ACE2), assisted by a protein spike (S). Afterward, it releases the virus genome material into the host cell (Muchtaridi et al., 2020).

In case of drug use, it should also be considered that people may suffer from drug‐related adverse effects, including gastric irritation, ulceration, angioedema, hepatic headache failure, hemolytic anemia, hyperglycemia, and immunodeficiency‐related problems, as well as others (Shahzad et al., 2020). Therefore, scientists are looking for new antiviral formulations. Today, various bioactive compounds with definite regulating effect on the immune system called immunomodulators have been identified. Many bioactives have been applied in the therapy of bacterial and viral infections (Labro, 2012). In this sense, natural compounds with high bioavailability and low cytotoxicity are the most efficient candidates (Muchtaridi et al., 2020). These compounds can prevent viral attachment and cell penetration effectively at the early stages of coronavirus infection, inhibiting the enzymes 3‐chymotrypsin‐like protease (3CLpro), papain‐like protease (PLpro), and ACE2 (Paraiso et al., 2020). Jo et al. (2020), for instance, showed that herbacetin, rhoifolin, and pectolinarin efficiently blocked the enzymatic activity of SARS‐CoV 3CLpro. Naringenin could also exert therapeutic effects against COVID‐19, preventing CoV‐encoded proteins, and ACE2 activity (Tutunchi et al., 2020). This review aims to report recent discoveries on the efficiency of bioactive compounds including polyphenols, glucosinolates, carotenoids, minerals, vitamins, oligosaccharides, bioactive peptides, essential oils, and probiotics as antiviral agents and recent findings on the effect of these compounds against coronaviruses as well as their mechanism of action have been compiled.

## 2 AN OVERVIEW OF CORONAVIRUS

**FIGURE 1:**
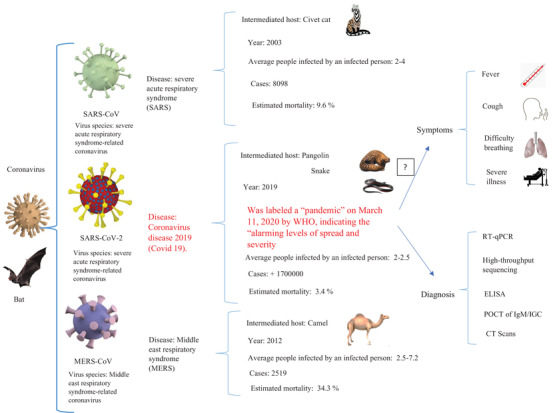
Overview of outbreaks associated with the important coronaviruses

The SARS‐CoV and the MERS‐CoV cases have confirmed that the coronaviruses are significant causes of severe respiratory disease, and more recently, COVID‐19 caused high levels of mortality (Figure 1). All types of coronaviruses are explained below in terms of their characteristics, mechanism, symptoms, and others.

### 2.1 MERS‐CoV (Middle East respiratory syndrome coronavirus)

Although the MERS‐CoV was diagnosed as the human respiratory pathogen in June 2012 in Saudi Arabia, as of April 15, 2020, globally a total of 2468 MERS‐CoV confirmed cases were reported (Khan et al., 2020). The MERS‐CoV, which is a betacoronavirus belonging to lineage C, is an enveloped virus with a single‐stranded RNA genome with a size of about 30 kb. The RNA genome acts as messenger RNA (mRNA), which plays the determining roles during the host cell cycle by being the initial RNA molecule for the infection cycle, the template for replication and transcription processes, and the substrate to be attached to the assembled viral particles (Bleibtreu et al., 2019). The genome of MERS‐CoV is organized like other species of coronavirus in which the first two thirds contains two overlapping reading frames that translate into the replication–transcription complex including 16 nonstructural proteins. The remaining one‐third genome is encoded to the four structural proteins and five accessory proteins that are not needed for the genome replication but may involve in virulence properties (Menachery et al., 2017).

The infection with MERS‐CoV is associated with the absence of specific clinical properties for differentiating it from other viral respiratory diseases (Degnah et al., 2020). An extreme variation for the clinical features of MERS‐CoV infection is observed; while 14%–80% of cases do not show any symptoms, others may present a flu‐like syndrome, pneumonia, and acute respiratory distress syndrome (ARDS). Fever (77%), cough (90%), and dyspnea (68%) are reported as the three most common symptoms, but several other secondary symptoms such as sputum production (40%), odynophagia (39%), and myalgia have been observed for the infection with MERS‐CoV (Baharoon & Memish, 2019). Also, the infection with MERS‐CoV has been significantly more associated with diarrhea compared with other acute respiratory conditions (Garbati et al., 2016). The average of crude fatality rate for the MERS‐CoV is 35% and 20% among primary cases and secondary cases, respectively (Alfaraj et al., 2019). The age of above 60 years, male gender, diabetes mellitus, chronic lung and chronic renal diseases, and progressive lymphocytopenia are among major contributing factors in poor results regarding MERS‐CoV infection (Degnah et al., 2020).

In high‐risk courtiers, healthcare facility transmission has been the major cause of MERS‐CoV infection, which is associated with the challenges in measuring the infection control as well as the late and nonefficient isolation of suspected cases. The control of infection in camels and the prevention of transmission from camel to human are the main preventive measures for controlling MERS‐CoV (Baharoon & Memish, 2019). The dromedary camels are the only confirmed animals that have been acting as the intermediate host for infection of human with MERS‐CoV. The close contact with camel (in terms of their respiratory droplets, saliva, organs during slaughtering), as well as the consumption of their products (such as milk and unprocessed meat), can lead to the infection (Kandeil et al., 2019). MERS‐CoV can poorly be transmitted from human to human, and that is why the new MERS‐CoV cases are predominantly restricted to the Arabian Peninsula and outbreaks outside that region are generally limited (Baharoon & Memish, 2019).

### 2.2 SARS‐CoV (severe acute respiratory syndrome)

SARS‐CoV is the causative agent of a sudden respiratory outbreak that occurred during 2002–2003, which belongs to the genus betacoronavirus of the family Coronaviridae. The first SARS‐CoV case was detected in late 2002 in Guangdong Province, China, followed by the rapid spread of disease, resulting in a SARS‐CoV outbreak in Hong Kong (in mid‐February 2003), and other outbreaks in 19 different countries, in which a total of 8,605 individuals became infected and 774 deaths were reported (Chow et al., 2003).

The palm civets, raccoon dogs, and horseshoe bats have been announced as the hosts of SARS‐like CoVs. However, it is demonstrated that the horseshoe bats can be considered as the only reservoir hosts because they are widely distributed and are very mobile mammals with the previously proven role as the hosts of emergent RNA viruses (García‐Salido et al., 2020a, 2020b). In studying the potential relationship between SARS‐CoV isolated from animals and humans, a 29‐nucleotide deletion was observed for the SARS‐CoV strain isolated from humans (rather than the isolate from civet) which indicated the one‐way transmission of virus from animals to humans (Giannis et al., 2020). Later, the genomic comparisons demonstrated that the recombination of SARS‐CoV between viruses isolated from human and animal or between various animal viruses was unlikely, which ruled out natural or laboratory chimerism and indicated that the SARS‐CoV was probably a zoonotic virus (da Silva et al., 2020).

The transmission through mucosal surfaces (with virus‐laden body fluids), which are the primary respiratory secretions, is demonstrated for the transmission of the SARS‐CoV from human to human. Large droplets of coughing and sneezing by a projection range of 1.0 m may have contributed into the airborne transmission of SARS‐CoV (Tong, 2005).

The clinical features of SARS‐CoV are associated with three phases—phase 1: the upper respiratory viral replication and viremia; phases 2 and 3: the lower respiratory tract viral replication; and phase 3: critical pulmonary injury (due to virus alone or in conjunction with immunological damage). SARS‐CoV could cause a high incidence of ARDS and respiratory syndrome, resulting in a high rate of death, even in healthy young individuals (Lin et al., 2005).

The rate of fatality for 138 hospitalized infected individuals with SARS‐CoV was 4.3% (Wang et al., 2020). However, the fatality rate for the large number of undetected mild infection cases could be below 1% (or even below 0.1%) (Wu et al., 2020).

Clinically, SARS‐CoV infection is characterized by fever, followed by respiratory symptoms that can potentially lead to progressive respiratory failure (Achak et al., 2020). In most cases, for a specific coronavirus, only the cells of natural host species and a few closely related species can be infected, but the SARS‐CoV has shown a high capacity in infecting diverse cell cultures (Giannis et al., 2020). The cell line of human colorectal adenocarcinoma, African green monkey, and kidney cells could be infected only after 2–3 days with SARS‐CoV, which clearly indicates its massive cytopathic effect (CPE) (Wang et al., 2020).

### 2.3 COVID‐19 (coronavirus disease)

The coronavirus disease (Covid‐19) is a pathogenic viral infection caused by SARS‐CoV‐2, and rapidly spread around the world. The World Health Organization declared the outbreak a Public Health Emergency of International Concern on January 30, 2020, followed by raising its global risk assessment to “Very High” on February 28, 2020. Also, later, on March 11, 2020, Covid‐19 outbreak was labeled a “pandemic,” which shows the “alarming levels of spread and severity” for this crisis.

As of November 24, 2020, over 58.9 million cases have been reported in more than 200 countries and territories, resulting in more than 1.3 million deaths, a death toll that is far beyond any other health crisis in modern history. The severity of public‐health crisis associated with the Covid‐19, such as the draconian containment efforts—quarantines, lockdowns, transportation bans, and restrictions on public assembly—has been producing a significant shock affecting the lifestyle of people and economy of many countries around the globe, which undoubtedly would be considered to be one of the biggest disasters facing humankind in modern history.

The biggest crisis associated with this coronavirus is its highly transmittable capacities, which challenged the majority of countries significantly. SARS‐CoV‐2 has shown the basic reproduction number (R0) of spread from person to person of about 2.6, indicating the existence of an exponential rate in its infection growth (Runfeng et al., 2020).

Although COVID‐19 is related to the SARS‐CoV and MERS‐CoV, COVID‐19 presents several specific pathogenetic, clinical, and epidemiological characteristics that have not been completely understood to date (Zhao et al., 2020). The genomic analysis revealing the phylogenetically relatedness of SARS‐CoV‐2 to SARS‐like bat viruses (88% identity) indicates that most probably bats have been the primary reservoir (Hamid et al., 2020). Although the intermediate source of origin and transfer from bats to humans is not known for now, the rapid transmission from human to human has been confirmed extensively (Shereen et al., 2020). The reproduction number for COVID‐19 is estimated to be in the range of 2.24 to 3.58 (Zhao et al., 2020). The direct contact or droplets from coughing or sneezing from COVID‐19–infected persons are the main causes of person‐to‐person transmission of COVID‐19. Also, there is no confirmed information regarding the potential transmission of this virus from mother to child (Rothan & Byrareddy, 2020).

The incubation period of infection with COVID‐19 is about 5.2 days. Although COVID‐19 has some similarities in its symptoms with other betacoronavirus—fever, dry cough, dyspnea, and ground‐glass opacities on chest CT scans—it presents several unique clinical features (Zhao et al., 2020). The COVID‐19 infection can be differentiated through targeting the lower airway, which can be clear through tracing the upper respiratory symptoms (such as rhinorrhea, sneezing, and sore throat) (Mulangu et al., 2019). Also, COVID‐19 develops intestinal symptoms like diarrhea, but low percentage of patients infected with MERS‐CoV or SARS‐CoV had diarrhea (Huang et al., 2020; Rothan & Byrareddy, 2020). Pneumonia in lungs and multiorgan failure are the severe complications for the COVID‐19.

### 2.4 Immune dysregulation/responses to coronavirus

The immune system plays an important factor in the severity of the pathogenesis of COVID‐19. Immune response is crucial to control and defense coronavirus infections. Dysregulation in the immune system can lead to an unappropriated local and systemic immune responses and subsequently the rapid spread of the virus (Tahaghoghi‐Hajghorbani et al., 2020). SARS‐CoV‐2 infection impaired cellular immunity by reducing the activated T‐cell markers, increasing expression of late activation markers including CD25 and PD‐1 in both CD4^+^ and CD8^+^ T cells, reduction in the lymphocyte number, and enhancing proinflammatory cytokines and even cytokine storm (Tahaghoghi‐Hajghorbani et al., 2020; Yang et al., 2020a). Moreover, it is believed that dysregulated host immune response and cytokine storm are correlated with disease severity and poor prognosis during SARS‐CoV and MERS‐CoV infection (Vafaeinezhad et al., 2021). Blot et al. (2020) investigated the immune response and results between non‐COVID‐19 and COVID‐19 patients with severe pneumonia. They reported that COVID‐19 patients had higher plasma granulocyte–macrophage colony‐stimulating factor (GM‐CSF) and C–X–C motif chemokine ligand 10 (CXCL10). These cytokines could represent the dysregulated immune response in severe COVID‐19, as well as promising therapeutic targets. In another study, Qin et al. (2020) observed that severe cases had lower lymphocyte counts, higher leukocyte counts and neutrophil–lymphocyte ratio, as well as lower percentages of monocytes, eosinophils, and basophils.

## 3 ANTIVIRAL ACTIVITY OF DIFFERENT BIOACTIVES

In recent times, extensive investigations have been carried out to explore the antiviral activity of different bioactive compounds from natural products, such as plants, fruits, vegetables, grains, fish, and meat, as a prospective source of different bioactives. Moreover, many of the natural products have polypharmacology or a promiscuous mechanism of action. The polypharmacology of natural products might enable them to surpass the use of the traditional single‐target drugs in terms of efficiency (Ho et al., 2018). In addition, the antiviral effect could be affected by the synergistic or additive effect from multiple components. For example, carrageenan and griffithsin combinations showed synergistic activity against SARS‐CoV‐1 and 2, including against recent SARS‐CoV‐2 mutations (Alsaidi et al., 2021). In this section, we discuss a wide range of bioactives with antiviral actions against coronavirus (COVID‐19), as well as their potent potential for further application in clinical practices.

### 3.1 Polyphenols

#### 3.1.1 Foods rich in polyphenols

Polyphenols are the main compounds with antioxidant activity present in plants (up to 90% of total dietary antioxidant capacity) (Saura‐Calixto et al., 2010). Most plant foods, including fruits, vegetables, cereals, legumes, nuts, and cocoa, as well as beverages originating from plants (e.g., tea, coffee, herbal infusions), contain an abundant and near‐ubiquitous number of various polyphenols. Fruits such as grapes, plums, blueberries, blackberries, cranberries, red raspberries, apricots, blackcurrants, cherries, apples, pears, nectarines, and citrus are rich sources of flavanols (Arts et al., 2000; Nile & Park, 2014; Zhou et al., 2020). Cruciferous vegetables, leafy green vegetables, and allium vegetables have all been known as rich sources of polyphenolic compounds (Steinmetz & Potter, 1996).

The type of phenolic compounds found in each plant or a variety of plants is also different. For example, catechin and epicatechin are the major potent flavanols found in fruits, whereas epigallocatechin and epigallocatechin gallate (EGCG) exist predominantly in tea (specifically, green tea), grapes, and some seeds of leguminous plants (Arts et al., 2000; Rashidinejad et al., 2016, 2017). Nevertheless, only a minor part of dietary polyphenols are absorbable in the human's small intestine, and the rest pass to the colon and interact with colonic microbiota and are converted to fermentable substrates and a nondigestible fraction (Saura‐Calixto, 1998; Scalbert & Williamson, 2000).

#### 3.1.2 Antiviral activity of polyphenols

So far, numerous epidemiological studies have confirmed the health‐promoting effects of polyphenols, originating from their antioxidant properties and the prevention of the impairment caused by oxidative stress in certain biomolecules (e.g., nucleic acids and proteins) (Chang et al., 2016; Fraga et al., 2019; Liu et al., 2008; Wichansawakun & Buttar, 2019). Oxidative stress is the result of the elevated intracellular levels of reactive oxygen species (ROS), which are by‐products of aerobic metabolism and include the superoxide anion (O_2_^−^), hydrogen peroxide (H_2_O_2_), and hydroxyl radicals (OH·), and can damage lipids, proteins, and DNA. Some specific mechanisms for the reaction of polyphenols in the body include their interaction with transcription factors, enzymes, and some receptors. Interactions with proteins, which depend upon the modification of enzymatic activities, transcription factors binding to the particular sites in DNA, and receptor–ligand binding that result in a biological effect (depending upon the protein function), are examples of the specific effective mechanisms of polyphenols (Ramassamy, 2006).

Although several studies have already shown that protease inhibitors such as polyphenolic compounds can be very effective in controlling virus‐induced infections (Liu et al., 2008, 2020; Mohammadi & Shaghaghi, 2020; Shaghaghi, 2020; Yang et al., 2020b), so far, there is not enough evidence from in vivo studies reporting the associations between polyphenols and downregulation of ACE2 expression related to COVID‐19. Nevertheless, it has recently been reported that polyphenols may interact with SARS‐CoV‐2 viral proteins and the corresponding cellular targets (Paraiso et al., 2020). Therefore, the possible modulation of immune response by these natural bioactive compounds can be considered as a beneficial aspect toward protection of human body against COVID‐19. Paraiso et al. (2020) showed the effect of polyphenols on various steps of the life cycle of SARS‐CoV‐2. Based on this theory, phenolic compounds such as resveratrol, curcumin, and emodin can potentially inhibit binding of SARS‐CoV‐2 spike protein to ACE2 receptor, by which the viral entry into the host cell is prevented. This, in turn, can inhibit the viral RNA replication and protein processing (Paraiso et al., 2020).

The use of polyphenolic compounds for the prophylaxis and treatment of COVID‐19 has also been reported in a recent study (Mhatre et al., 2021) that reviewed antiviral activities of major polyphenols in both green and black tea. Catechins from green tea, especially EGCG, and theaflavins from black tea, especially theaflavin‐3,3′‐digallate (TF3), have shown a strong binding with receptors responsible for COVID‐19 (Mhatre et al., 2021). Turmeric and its most active bioactive (i.e., curcumin) have been suggested to be feasibly tested as preventive and/or treatment options for COVID‐19 patients (Verma, 2020). This is supported by the evidence related to the action of curcumin at an early step in SARS‐CoV‐2 infection via the inhibition of its entry into the host cell, besides the effect on the inhibition of the virus replication in human cells. In addition, phenolic compounds such as curcumin with high antioxidant activity are known as potent anti‐inflammatory agents (Chainani‐Wu, 2003), meaning that this can be a promising approach toward the relief of COVID‐19 symptoms.

Antiviral activity of the polyphenols from both green and black teas in prophylaxis and treatment of COVID‐19 has been reviewed very recently (Mhatre et al., 2021).

Other polyphenols such as those extracted from *Isatis indigotica*, *Houttuynia cordata*, Chinese rhubarb, litchi seeds, *Scutellaria baicalensis*, *Galla chinensis*, and *Veronicalina riifolia* are also suggested to show inhibition of SARS‐3CL^pro^ activity, the cellular entry of SARS‐CoV, and the 3a ion channel of Coronavirus SARS‐CoV and HCoV‐OC43, as well as the prevention of the early stage of HCoV‑22E9 infection (Yang et al., 2020). The antiviral activity of polyphenolic compounds such as flavonoids against other viruses has also been reported. For example, anti‐influenza virus activity of flavonoids from the medicinal plant *Elsholtzia rugulosa* has been investigated (Liu et al., 2008). The in vitro antiviral assay using a CPE reduction method showed that five active polyphenolic compounds in *Elsholtzia rugulosa*, including apigenin, luteolin, apiin, galuteolin, and luteolin 3′‐glucuronyl acid methyl ester, presented anti‐influenza virus activity. Among these active compounds, apigenin and luteolin were reported as the most potent flavonoids against influenza virus (H3N2) (IC_50_ values of 1.43 and 2.06 μg/mL, respectively) (Liu et al., 2008). This confirms that systematic research toward the identification of effective natural/herbal formulations containing polyphenolic compounds that could reduce the ACE2 expression on epithelial cells may result in discovering novel preventive measures for COVID‐19. In addition, Shikonin is the root extract of *Lithospermum erythrorhizon* Sieb. et Zucc. (Boraginaceae), widely used in traditional Chinese medicine for its antioxidant, anti‐inflammatory, antithrombotic, antimicrobial, and wound‐healing effects (Andújar et al., 2013). The crystal structure of main protease in complex with Shikonin structure highlights a new mode of binding, and may serve as an invaluable resource to improve the design of novel antiviral drugs (Li et al., 2021).

El‐Missiry et al. () reported that polyphenols could be potential nutritional adjuvants for targeting COVID‐19. They stated that health‐promoting effect of these natural compounds on COVID‐19 might mostly be due to strengthening the body's anti‐inflammatory and antioxidant defenses against viral infection. However, some other mechanisms such as targeting virus proteins and/or blocking cellular receptors can also be effective in preventing the entry of the virus in the host cells and its replication (El‐Missiry et al., 2021).

Therefore, although it is still too early to have robust in vivo evidence for the efficacy of polyphenols against COVID‐19 pandemic, the primary investigations so far can pave the way for systematic and advanced experimental research for the investigation of the efficacy of polyphenolic compounds from natural sources for the prevention and/or treatment of COVID‐19. Nevertheless, it should be noted that numerous factors can affect the antioxidant properties of polyphenols, which should also be considered when studying their antiviral activity. These include the subsequent metabolism and absorption in the digestive tract, which governs their biological characteristics (Tarko et al., 2013). Food polyphenols (in their native form) mainly exist in forms of polymers, esters, and glycosides. These compounds cannot be absorbed as such, meaning that they need to be hydrolyzed by endogenous enzymes and/or microflora enzymes in the digestive tract (Williamson & Clifford, 2010). The nature of the food matrix, pH, the gastrointestinal environment, and the presence of bile salts can also significantly affect the metabolism and bioefficacy of polyphenols (Manach et al., 2004), so delivery/encapsulation systems can be an effective strategy.

### 3.2 Glucosinolates

Glucosinolates are a large group of sulfur‐containing glucosides synthesized as secondary metabolites in plants. Glucosinolates are anionic, nonvolatile, water‐soluble and thermostable compounds. These compounds are found in cruciferous plants mainly *Brassicaceae* families such as broccoli, Brussels sprout, cabbages, and cauliflower. The main glucosinolates that are presented in *Brassica* vegetables include sinigrin, gluconapin, glucobrassicanapin, glucoiberverin, glucoiberin, glucoraphanin, glucoerucin, progoitrin, napoleiferin, glucotropaeolin, gluconasturtin, glucobrassicin, 4‐methoxyglucobrassicin, and neoglucobrassicin (Horbowicz, 2003).

Glucosinolates and their breakdown products are responsible for the pungent flavor in these vegetables. When these vegetables are consumed without processing, the myrosinase enzyme (also known as β‐thioglucosidase) that is present in these vegetables can hydrolyze glucosinolates in the small intestine to different compounds such as isothiocyanates, indole‐3‐carbinols, nitriles, oxazolidine‐2‐thiones, and sulforaphane. After processing the vegetables, such as by cooking, the myrosinase enzyme is inactivated and glucosinolates are broken down by the enzyme of microbiota in the colon to other compounds such as isothiocyanates and glucose. Isothiocyanates are absorbed from the colon and small bowel and can hinder apoptosis and mitosis in human cancer cells especially lung cancers and the alimentary tract (Barba et al., 2016; Horbowicz, 2003; Johnson, 2002; Saladino et al., 2017). Verhoeven et al. (1996) concluded from a meta‐analysis study that brassica vegetables can protect against different cancers such as lung, rectum, stomach, and colon.

Glucosinolates have antioxidant, anticarcinogenic, and antimicrobial properties and can also be used as natural agents for food preservation (Saavedra et al., 2010). The antimicrobial activity of glucosinolates and their derivatives has been proved in many studies. These compounds have high antimicrobial activity against different bacterial and fungal species (Borges et al., 2015; Dias et al., 2012; Saladino et al., 2017). Some of the studies have also confirmed the antiviral activity of glucosinolates (Table 1).

**TABLE 1:** Selected studies about the antiviral activity of the glucosinolates

Bioactive compound	Antiviral activity against	Model	Key outcomes	References
*Isatidis Radix* (a traditional Chinese medicine)	Influenza A virus (H1N1)	In vitro (on Madin–Darby canine kidney (MDCK) cells) and in ovo (on embryonated eggs)	‐ *Isatidis Radix*–derived glucosinolate isomers (epiprogoitrin and progoitrin) and their breakdown products (epigoitrin and goitrin) indicated antiviral activity against influenza A virus (H1N1) without toxicity.	(Nie et al., 2020)
Broccoli seeds with high sulforaphane content	influenza A/WSN/33(H1N1) virus	In vitro (on MDCK cells)	‐ Sulforaphane is an isothiocyanate that produced by hydrolyzing the glucoraphanin‐rich broccoli. ‐ Extracted sulforaphane from broccoli seeds showed antiviral activity against influenza A.	(Z. Li et al., 2019)
*Isatis indigotica*	Influenza A virus (H1N1)	In vitro (MDCK cells and human alveolar epithelial cell line (A549) In vivo (on the mouse)	‐ Epigoitrin as a natural alkaloid from *Isatis indigotica* can make a protection against influenza virus. ‐ Epigoitrin can decrease viral duplications in the lungs. ‐ Epigoitin can increase mitochondria antiviral signaling.	(Luo et al., 2019)
*Brassica juncea* (also known as brown mustard) extract	Influenza H1N1 virus A/NWS/33	In vitro (on MDCK cells)	Ethanol extract of *Brassica juncea* reduced nearly 3 Log of tissue culture infective dose at 50%/25 μL.	(Bae et al., 2019)
*Brassica juncea* extract	Influenza A virus (H1N1)	In vitro (on MDCK cells)	‐ The glucosinolate compounds of *Brassica juncea* extract are sinigrin, gluconapin, and glucobrassicin ‐ Subcritical water extract (SWE) of *Brassica juncea* can be used as a food supplement for prevention of influenza viral infection. ‐ 0.28 mg/mL of *Brassica juncea* SWE was added to nonfat milk and indicated 39.62% antiviral activity. ‐ The viability of MDCK cells that were infected with influenza virus was decreased up to 50% by the addition of 0.5 mg/mL of *Brassica juncea* SWE to culture medium.	(N.‐K. Lee et al., 2014)
Maca (*Lepidium meyenii*)	Influenza A virus (H1N1) and influenza B virus	In vitro (on MDCK cells)	‐ The methanol extract of maca showed antiviral activity against influenza A and B. ‐ The antiviral activity may be attributed to glucosinolates, active isothiocyanatesm, alkaloids, flavonoids and saponins, essential fatty acids and benzoyl derivatives.	(Del Valle Mendoza et al., 2014)
*Isatis indigotica* root *(Isatidis Radix)*	SARS coronavirus	In vitro (using cell‐free and cell‐based cleavage assay)	‐ *Isatis indigotica* root‐derived compounds and the water extract of *Isatis indigotica* showed inhibitory effect on the SARS coronavirus 3C‐like protease ‐ Sinigrin as a glucosinolate compound in *Isatis indigotica* root showed high efficacy with IC_50 _= 217 μM in blocking the cleavage processing of 3C‐like protease	(Lin et al., 2005)

Other sources such as *Isatidis Radix* (a traditional Chinese medicine belonging to the family Cruciferae) as an herbal remedy has glucosinolate in its structure and have shown antiviral activity (Xie et al., 2011; Zhang et al., 2013). There are three main active ingredients in *Isatidis Radix*, alkaloid compounds that mainly consist of epigoitrin (one of the degradation products of glucosinolate isomers), organic acids such as salicylic acid, and total lignans represented by clemastanin (Zuo et al., 2007). It is reported that *Isatidis Radix* has high efficacy against respiratory syncytial virus (RSV)‐induced pneumonia; however, its mechanism is not clear. Xu et al. (2019) reported that active ingredients of *Isatidis Radix* can show antiviral activity alone or in combination synergistically.

### 3.3 Carotenoids

Carotenoids, a wide range of organic pigments (including lycopene, α‐carotene, β‐cryptoxanthin, β‐carotene, zeaxanthin, and lutein), are presented broadly in nature (Rehman et al., 2020b). Their wide‐ranging bioactivities, including antiviral, antidiabetic, anti‐inflammatory, antiaging, cardioprotective, anticancer, have gained much acceptance and extremely supported via a number of studies (Ashraf et al., 2020; Giuffrida et al., 2020). Carotenoids belong to the diverse family of organic pigments, including red, orange, and yellow, that have the capability to absorb the light around 500 nm. They are not synthesized by humans; however, their needs are only fulfilled by consuming diets. The most abundant sources of more than 40 carotenoids are plants, photosynthetic organisms, flowers, fruits, algae, and few yeasts. Because of having diverse structural arrangements, they retain plenty of numerous biological functions that can maintain human health (Britton, 2020). The defensive properties of carotenoids against several viruses that can cause cancer, eye diseases, heart disease, and microbial infection have successfully been proposed in recent studies. More interestingly, the leading role of carotenoids being antiviral, antioxidant, and potent regulators of the immune response system has been documented (Mozaffarieh et al., 2003; Sesso et al., 2003). Lycopene is known to be one of the major carotenoids in the diet that offers red color mostly to the vegetables and fruits. Dietary intake of tomatoes, containing massive amount of lycopene, is considered the foremost tool to reduce the risks of chronic diseases (Ashraf et al., 2020). Therapeutically, antioxidant attributes of lycopene can provide protection to the cells from antagonistic results caused by any kinds of inflammation. As an example, it has been reported that lycopene plays a vital role in defeating airway inflammation caused by rhinovirus through decreasing the emancipate of interleukin‐6 and interferon‐gamma‐induced protein (Saedisomeolia et al., 2009). β‐Carotene is a carotenoid found in many red and orange fresh fruits and vegetables and has strong antioxidant property. Vitamin A–rich foods include carrots, onions, peas, squash, and spinach. Various studies have shown that β‐carotene decreases hepatosteatosis induced by HCV by inhibiting viral RNA replication. Provitamin A has a strong role in decreasing ROS and preventing the development of carcinoma hepatocellular progression caused by the hepatitis viruses HBV and HCV (Yadav et al., 2002). In another study, the powerful therapeutic antiviral activity of the extracted carotenoids was explored in contradiction of HBV and HCV by preventing HBV DNA‐dependent DNA polymerase and HCV NS5B polymerase, which ultimately overwhelms HBV and HCV replication (Hegazy et al., 2020).

Lutein is one of two major carotenoids found in dark‐green vegetables, such as kale, spinach, and broccoli. Foods such as egg yolk, peppers, and grapes are also good sources of lutein. Antiviral activity of lutein against hepatitis B has also been reported, as it inhibits transcription of the virus (Pang et al., 2010). The main active compound of turmeric is curcumin. Curcumin has strong anti‐inflammatory properties, and various animal studies confirmed that it has the potential to improve immune function (Rehman et al., 2019). Astaxanthin has well‐documented anti‐inflammatory and immune‐stimulating effects (Rehman et al., 2020a). Dietary supplementation with astaxanthin significantly increased levels of antioxidant enzymes such as glutathione peroxidase and catalase superoxide dismutase in rats (Ambati et al., 2014; Rao et al., 2013).

Astaxanthin also improved antibody production, as testified in older animals, signifying that this carotenoid supplementation could be more useful in restoring humoral immune response (Okai & HigashiOkai, 1996). Now it is clear that many carotenoids found in foods promote the immune system and have strong antioxidant activity; some have even shown direct antiviral activity. The mechanisms of action and molecular targets are still unidentified, so comprehensive studies of these compounds are required to develop them as future therapeutic drugs for the treatment of COVID‐19.

### 3.4 Minerals

Human diet consists of a wide brand of minerals (micronutrients) such as Fe, Zn, and Ca, and they have therapeutic potential; however, they are playing a crucial role in boosting the immune system and preventing the viral infections, as well as preserving the homeostasis process in human body. Additionally, the abovementioned micronutrients have successfully been investigated by numerous scientists in order to explore their pharmacological attributes, including antiviral, anti‐inflammatory, anticancer, antioxidant, and antidiabetic characteristics (Gharibzahedi & Jafari, 2017).

Collectively, all micronutrients are considered very necessary for several reasons, specifically owing to their and antiviral and anti‐inflammatory properties. Zinc is a trace element essential, aids in boosting the immune system, supporting the body growth, and healing the wounds. To date, it is reported that deficiency of Zn leads toward severe immune dysfunctions (Wessels & Rink, 2020). Interestingly, numerous studies have recently reported a great loss in human senses, such as smelling and tasting senses in the start of COVID‐19–infected patients (Keyhan et al., 2020; Lechien et al., 2020). According to the previous literatures based on zinc deficiency, it has proved that loss in taste could be due to zinc COVID‐19–infected patients, and supplementation of zinc has displayed outstanding results in curing the loss of taste (Doty, 2019; Yagi et al., 2013). Collectively, the loss in taste and smell of COVID‐19–infected patients may be associated with zinc deficiency. Obviously, zinc is an effective inhibitor of several RNA viruses like SARS‐CoV (Velthuis et al., 2010). Such inhibiting approach of zinc in the repetition of COVID‐19, it is proposed that zinc may have a lot of positive consequences for COVID‐19–infected patients meanwhile zinc supplements are easily existing in the world markets.

Zinc deficiency significantly affected the ability of our immune system to work properly, which resulted in an increased risk of infection, including pneumonia (Shah et al., 2016; Wang et al., 2020). Various studies have revealed that supplements of zinc may protect against respiratory tract infections, such as the common cold (Read et al., 2019). Around 2 billion people worldwide are affected by zinc deficiency, which is very common in older adults (Wessels et al., 2017). In a recent study, children with acute lower respiratory tract infections in various hospitals were given 30 mg of zinc per day that resulted in decreased total duration of infection and hospital stay duration by an average of two days in comparison with a placebo group (Rerksuppaphol & Rerksuppaphol, 2019). Although supplements of zinc exist in the market, the best way to get the benefits of this mineral is by including it in your daily diet. Experts recommend 11 mg of zinc per day for men and 8 mg for women during the flu and cold seasons. Dietary sources of zinc include meat, shellfish, chickpeas, lentils, beans, nuts, dairy, eggs, and whole grains. Selenium is an essential mineral for immune health. Selenium has been identified as a protective factor against some types of viruses, such as HIV (Baum et al., 2000).The best source of selenium is whole grains, yogurt, milk, meat, fish, shellfish, eggs, etc. Consumption of functional foods rich in zinc and selenium could contribute in reducing the COVID‐19 risks.

Copper, an imperative mineral, is equally important for both host and pathogen during viral infections. Copper has the capability in involving the bioactivities of following blood cells, including B cells, macrophages, and neutrophils natural killer (NK) cells (Raha et al., 2020). These blood cells have potent potential in terms of killing infectious microbes as well as have ability to produce specific antibodies in contradiction of pathogens (Iakovidis et al., 2011). Copper‐deficient individuals’ medical reports had showed an extraordinary vulnerability to infections accredited to the reduced quantity and imperfect activities of blood cell lines.

In addition, copper has the ability of destroying several viruses, including SARS‐CoV‐2 (Wazir & Ghobrial, 2017). Considering the rapid spread of COVID‐19 and since no drugs or vaccines provide 100% protection, it is important to boost the immune system to be able to fight against the COVID‐19. Based on accessible facts and figures, we assume that copper‐enriched foods can boost up immunity of humans.

### 3.5 Vitamins

Among all micronutrients in fruits and vegetables, vitamins, mainly vitamins A, B, C, D, and E, are responsible for immune reactions and have shown significant antiviral effects against the novel coronavirus (Calder et al., 2020). It has been proposed that the use of these vitamins could be sufficient to prevent and also treat the viral infections caused by SARS‐CoV‐2 (Gasmi et al., 2020). The water‐soluble and fat‐soluble vitamins with proven antiviral effects will be highlighted in the following subsections (Table 2).

**TABLE 2:** Selected studies about the antiviral activity of vitamins

Bioactive compound	Antiviral activity against	Model	Key outcomes.	References
Vitamin C	Common cold viruses	In vivo (clinical)	The subjects who received vitamin C had a 0.80‐fold lower risk of getting a common cold compared with the placebo group.	(Kim et al., 2020)
	Enterovirus/rhinovirus	In vivo (Clinical)	High dose of vitamin C caused a rapid resolution of lung injury in patients with virus‐induced ARDS.	(Fowler Iii et al., 2017)
	SARS‐CoV‐2 (COVID‐19)	In vivo (clinical)	Coadministration of vitamin C and quercetin may exert a synergistic antiviral effect in COVID‐19 patients.	(Biancatelli et al., 2020)
Vitamin B2	MERS‐CoV	In vivo (clinical)	Riboflavin and UV light significantly diminished the titer of MERS‐CoV to below the limit of detection in human plasma products which revealed the role of the vitamin in reducing the risk of transfusion and transmission of MERS‐CoV.	(Keil et al., 2016)
Vitamin B6	SARS‐CoV‐2 (COVID‐19)	In vivo (clinical)	Vitamin B6 supplementation may mitigate the symptoms of COVID‐19 via alleviating both the immune suppression and bolstering the endothelial integrity as well as preventing hypercoagulability.	(Desbarats, 2020)
Vitamin B9	SARS‐CoV‐2 (COVID‐19)	*In silico* (Molegro virtual docker version 6.0 software)	Results showed that folic acid could be utilized to inhibit the furin as an effective enzyme in proteolytic pathways could be useful in the management or prevention of COVID‐19 at the early stages of the respiratory disease.	(Sheybani et al., 2020)
Vitamin D	Rotavirus	In vitro (IPEC‐J2); in vivo (pig)	Vitamin D alleviated rotavirus infection through the TBK1/IRF3 signaling pathway via directly targeting TBK1.	(Y. Zhao et al., 2019)
	Influenza A virus	In vivo (clinical)	Vitamin D3 supplements reduced the incidence of influenza A in schoolchildren.	(Zhou et al., 2018)
	Influenza A and B virus	In vivo (clinical)	Vitamin D significantly reduced respiratory viral infection and the incidence of influenza by about 25%.	(Loeb et al., 2019)
Vitamin A	Bovine coronavirus	In vivo (calves)	Deficiency of vitamin A increases susceptibility to infectious disease in calves and low vitamin A diets may interfere the effectiveness of viral vaccines.	(Jee et al., 2013)
	IBV and reovirus (RV)	In vitro (chicken)	Infection with IBV and RV led to the acute respiratory disease in chickens and the infection was more serious in vitamin A–deficient chickens. This group showed a higher severity and frequency of the symptoms.	(West et al., 1992)
Vitamin E	Common cold viruses	In vivo (clinical)	Protective effect of vitamin E supplementation was observed on upper respiratory tract infections, especially the common cold.	(Meydani et al., 2004)

#### 3.5.1 Water‐soluble vitamins

B vitamins are water‐soluble vitamins commonly found in poultry, fish, meat, potatoes, meat, egg, nuts, legumes, whole grains, seaweed, etc. (Chowdhury, 2020). B vitamins work as a part of coenzymes in the human body. Each B vitamin has its own special functions. For instance, vitamin B1 or thiamine can modulate the immune system function and is able to decrease the risk of type 2 diabetes, aging‐related disorders, cardiovascular diseases, mental disorders, kidney disease, cancer, and neurodegenerative disorders (Mikkelsen & Apostolopoulos, 2019). Because the antibodies, mainly T cells, are necessary to suppress the SARS‐CoV‐2 virus, vitamin B1 deficiency can potentially lead to insufficient antibody responses, which can subsequently result in severe symptoms. Therefore, adequate thiamine levels would be a critical factor to achieve the proper immune responses during the coronavirus infection (Shakoor et al., 2021). Additionally, the symptoms of altitude sickness and pulmonary edema, which are commonly prevented by prescription of acetazolamide through the inhibition of carbonic anhydrase isoenzymes, and further elevation of oxygen levels have been observed in COVID‐19 patients. Thiamine also acts as an inhibitor of carbonic anhydrase isoenzyme. Therefore, giving high doses of thiamine at early stages of COVID‐19 could decrease hospitalization and limit hypoxia (Shakoor et al., 2021). Vitamin B2 or riboflavin is necessary for the energy metabolism of cells (Zhang & Liu, 2020). It has been reported that vitamin B2 along with UV light decreased the concentration of MERS‐CoV in human plasma (Keil et al., 2016). Riboflavin and UV light result in an irreversible damage to DNA and RNA, disabling microbial pathogens to replicate (Shakoor et al., 2021).

Vitamin B3 or niacin/nicotinamide is one of the components of nicotinamide adenine dinucleotide (NAD) and NAD‐phosphate, which are vital through chronic systemic inflammation. NAD^+^ is released as a coenzyme at the early stages of inflammation in different metabolic pathways, and its enhanced levels are required to treat a broad range of pathophysiological states. The immunomodulatory properties of NAD^+^ may result in decreased proinflammatory cytokines, TNF‐α, IL‐1β, and IL‐6 (Boergeling & Ludwig, 2017). According to the recent evidence, targeting IL‐6 in patients with COVID‐19 might be helpful to control the inflammatory storm (Liu et al., 2020a). Additionally, nicotinamide can decrease viral replication and strengthen the body's immune system. So, niacin would be a good adjunct treatment for COVID‐19 patients (Shakoor et al., 2021). Moreover, vitamin B3 can significantly inhibit the neutrophil infiltration into the injured lungs with a considerable anti‐inflammatory effect. Nevertheless, this treatment resulted in the development of hypoxemia (Jones et al., 2015).

There are limited studies on vitamin B5 or pantothenic acid on the immune system. However, it has been reported that vitamin B5 may decrease inflammation (Mikkelsen & Apostolopoulos, 2019). Furthermore, vitamin B6 or pyridoxine is required for protein metabolism and participates in more than 100 reactions in different tissues as well as immune responses to viral infections, and its deficiency may lead to immune dysregulation. So, vitamin B6 is an ideal supplement for virus‐infected patients to improve their immune system and could be considered as a basic option for the treatment of the novel coronavirus (Zhang & Liu, 2020). A recent preprint has suggested that vitamin B6 supplementation relieves the symptoms of COVID‐19 by regulation of immune responses, reduction of proinflammatory cytokines, maintenance of endothelial integrity, and prevention of hypercoagulability (Desbarats, 2020). Due to the approved effect of vitamin B6 on the upregulation of IL‐10 as an immunosuppressive and anti‐inflammatory cytokine, it may dampen the inflammation and cytokine storm caused by the COVID‐19 virus (Shakoor et al., 2021).

Vitamin B9 or folic acid/folate is a vital vitamin for protein and DNA synthesis and immune responses. It has recently been noted that vitamin B9 can prevent SARS‐CoV‐2 cell entry and viral turnover. So, it could be a beneficial agent to suppress the viral infection (Sheybani et al., 2020). Vitamin B12 or cobalamin is vital for red blood cell synthesis, cellular growth, DNA synthesis, and nervous system health. However, the symptoms of vitamin B12 deficiency are similar to the infection of SARS‐CoV‐2 such as hyperhomocysteinemia, increased oxidative stress, coagulation cascade activation, and pulmonary vasculopathy (Sabry et al., 2020). Recently, some clinical studies have reported the effect of vitamin B12 supplements on the decrement of lung damage, severe symptoms, and the need for intensive care support (dos Santos, 2020; Tan et al., 2020a). Vitamin B12 is also vital to support a healthy gut microflora, which has an essential role in the function and development of both adaptive and innate immune systems. This could be fundamental in COVID‐19 cases with gut microflora dysbiosis (Zuo et al., 2020). A combination of vitamin B12/magnesium/vitamin D in older COVID‐19 patients was significantly associated with a decreased clinical deterioration implicating oxygen support or intensive care. However, further randomized controlled trials are needed to find novel combinations with more efficient effects in ameliorating the severity of symptoms in COVID‐19 patients (Tan et al., 2020b).

Vitamin C or ascorbic acid is another water‐soluble vitamin, and its commontan sources are citrus fruits, kiwi, yams, broccoli, strawberries, and melons. The recommended intake dosage of vitamin C for adults is 60–90 mg/day (Chowdhury, 2020). Vitamin C is known for its essential role as an antioxidant and in immune functions, which can provide protection against coronavirus infection (Hemila, 2017). For instance, it has been reported that vitamin C enhanced the resistance of chick‐embryo ciliated tracheal organ cultures to infection by an avian coronavirus (Atherton et al., 1978). Another role of vitamin C is acting as an antihistamine agent to improve flu‐like symptoms, such as a running nose, sneezing, and swollen sinuses (Zhang & Liu, 2020). Additionally, human trials have revealed that vitamin C‐supplemented groups showed a lower incidence of pneumonia, which confirms that vitamin C may inhibit the susceptibility to respiratory tract infections (Hemila, 1997). Vitamin C exerts its antiviral characteristics via supporting the lymphocyte activity, enhancing the production of interferon‐α, decreasing inflammation, modulating cytokines, restoring mitochondrial function, and improving endothelial dysfunction. It has also been suggested that vitamin C might be directly viricidal (Biancatelli et al., 2020). High dose of vitamin C has also been successfully applied in the treatment of moderate to severe SARS‐Cov‐2 patients in China. Using 10–20 g of vitamin C per day led to a better oxygenation index in real time, and all of the patients were cured and discharged. Considering that high dosage of vitamin C is safe, researchers and healthcare professionals would better take this opportunity for its potential use in the treatment of Covid‐19 (Cheng, 2020).

#### 3.5.2 Fat‐soluble vitamins

Vitamin A with three active forms, such as retinal, retinol, and retinoic acid, is commonly found in liver, eggs, oily fish, carrots, orange fruits, fortified margarine, dairy products, tomato juice, green and yellow vegetables, and its recommended daily intake is 3000–5000 international unit (IU) for adults. Vitamin A has also been called an “anti‐infective” vitamin because its deficiency leads to an impaired immune system. Vitamin A supplementation decreased the symptoms and mortality of various infectious diseases (Zhang & Liu, 2020). It has also been reported that deficiency of vitamin A in diets might endanger the efficiency of vaccines made by an inactivated bovine coronavirus (Jee et al., 2013). Vitamin A and its derivatives can facilitate the modulation of innate immunity, barrier function, and enhancing the maturation and maintaining of NK cells such as dendritic cell and T helper 1 or 2 lymphocytes (Chowdhury, 2020). Many systematic reviews have supported that vitamin A could improve the symptoms of acute pneumonia and also enhanced the clinical responses and decreased the length of hospital stay (Hu et al., 2018). Overall, vitamin A could be a promising choice for the prevention of lung injuries and the treatment of novel coronavirus (Zhang & Liu, 2020).

Vitamin D is commonly supplied by sunlight, liver, eggs, fortified margarine and dairy products, and oily fish, and the recommended daily intake is 400–1000 IU for adults. The active form of this vitamin is 1,25‐dihydroxyvitamin D3 with approved immune‐regulatory properties. The involved mechanisms of immunomodulatory effects are complex. However, deficiency of vitamin D was associated with respiratory tract infections (Chowdhury, 2020). In a systematic review, it has been reported that the risk of pneumonia could be elevated with a deficiency of vitamin D (Zhou et al., 2018). Furthermore, several investigations and systematic reviews have exhibited that vitamin D can decrease the risk of infections in the respiratory tract (Autier et al., 2017; Bergman et al., 2013; Martineau et al., 2017, 2019). Three mechanisms have been proposed for this effect of vitamin D involving maintenance of tight junctions, induction of cathelicidins and defensins, which can decrease the rate of viral replication and reduce the cytokine concentration, which may produce inflammation and injures in the respiratory tract (Grant et al., 2020). Vitamin D impacts both adaptive and cellular immunity. However, 1,25(OH)2D3 can diminish the responses resulting from T helper type 1 (Th1) and can downregulate the activities of T cells, which may lead to lower inflammatory activities (Jeffery et al., 2009). Furthermore, vitamin D can increase cellular immunity by reducing the cytokine storm caused by viral infections. For this purpose, high concentrations of the active form of vitamin D (100–150 nmol/L) were preferred to get the most efficient result (Grant et al., 2020). Cytokine storm can be induced by the innate immune system causing the inflammatory and anti‐inflammatory responses in COVID‐19 cases. So, there is a direct relation between the function of vitamin D and the level of immunity in patients (Huang et al., 2020). As a preventive measure, it is proposed that people at risk of COVID‐19 take 10,000 IU/d of vitamin D3 for a few weeks to swiftly raise concentration of 25(OH)D, followed by a dose of 5000 IU/d. The target should be to elevate 25(OH)D concentrations up to 40–60 ng/mL. As a therapeutic measure, higher vitamin D3 dosage might be beneficial. However, large population investigations and randomized controlled trials should be organized to study these recommendations (Grant et al., 2020).

Vitamin E, including tocopherols and tocotrienols, is commonly found in plant oils (e.g., corn, soy, olive), seeds, nuts, and wheat germ. The recommended daily intake of vitamin E is 15–20 mg per day for adults (Chowdhury, 2020). Vitamin E is also significantly effective in the reduction of oxidative stress via blocking the free radicals because of its antioxidant characteristics (Galmes et al., 2018). It has been announced that there is a positive correlation between vitamin E and cellular immunity and vitamin E supplements can also improve the activity of helper T lymphocyte and enhance the vaccine responses (De la Fuente et al., 2008). Moreover, studies have confirmed that vitamin E supplements may help to decrease the risk and duration of respiratory tract infections (Chowdhury, 2020; Meydani et al., 2004; Zhang et al., 2019). Overall, it can be concluded that vitamins can be ideal supplements due to their immune‐augmenting roles for the prevention and treatment of coronavirus infections.

### 3.6 Polysaccharides and oligosaccharides

A clinical study in Wuhan, China, involving 41 patients with coronavirus infection revealed that intensive care patients had higher plasma levels of proinflammatory cytokines such as MCP1, MIP1A, IL‐2, IL‐7, IL‐10, IP10, GSCF, and TNF‐α than the other patients, which could be associated with Th1 cell responses (Suwannarach et al., ). Among all bioactives, various oligosaccharides and polysaccharides with proven health‐promoting and therapeutic effects have been introduced. Immunomodulatory properties and antiviral effect of poly‐ and oligosaccharides have been widely investigated. Poly‐ and oligosaccharides are valuable compounds with confirmed antiviral activity by stopping viral proliferation, binding to the receptors of the host cell, controlling adsorption of the virus, inhibiting the virus fusion to the host cell membrane, and regulating intracellular signals as well (Farshi et al., 2020). It has been proven that many mammalian viruses have developed to utilize glycans as a candidate for host cell receptors, and the probable association between coronavirus and glycans, which have been expressed on the surface of host cells, cannot be ignored. These phenomena can be used to design the glycans and specially oligo/polysaccharides with potent behavior as decoy receptors for the coronavirus (Walsh et al., 2020).

Polysaccharides are high‐molecular‐weight compounds extracted mostly from plants, algae, or animals. There are various bioactive polysaccharides with different sources, including cellulose, hemicelluloses (e.g., xylans, galactomannans and glucomannans), chitin, and chitosan, alginate, carrageenan, and lentinan (Bhatia et al., 2019). Some known polysaccharides such as chitosan, carrageenan, β‐glucan, *astragalus* polysaccharide, and fucoidan, have shown considerable antiviral activity (Bhatia et al., 2019; Chen et al., 2020; Huang et al., 2016). Particularly, sulfated polysaccharides can block the positively charged receptors on the surface of viruses and subsequently prevent the binding process to heparan sulfate proteoglycan that is placed on the cell surface, which may lead to inhibition of the entry process of the virus to the host cell. Nevertheless, the antiviral characteristics of polysaccharides not only depend on their charge density but also on their precise structural properties that are critical (Chen et al., 2020). Further, some of known antiviral polysaccharides are highlighted (Table 3).

**TABLE 3:** Selected studies about the antiviral activity of the oligo/polysaccharides

Bioactive compound	Antiviral activity against	Model	Key outcomes	References
N‐(2‐hydroxypropyl)‐3‐trimethylammonium chitosan chloride	Human coronaviruses (HCoV‐NL63, HCoV‐KU1, HCoV‐OC43, and HCoV‐229E)	In vitro (LLC‐Mk2 cells)	The chitosan derivative inhibited the interaction of studied coronaviruses with their receptor and thus blocked their entry into the cells.	(Milewska et al., 2016)
Chitosan	Avian influenza virus A (H5N2, H5N1, H5N2, H5N3)	In vivo (mice)	Chitosan can be a promising adjuvant candidate for inactivated influenza vaccines.	(Ghendon et al., 2009)
Lentinan	HIV	In vivo (HIV patients)	Lentinan qualifies as an ideal antiviral due to the stimulation of significant increase in CD4 levels in HIV‐infected patients.	(Gordon et al., 1995)
Astragalus membranaceus polysacharide	Gamma‐herpesvirus 4	In vitro (Raji cells)	The polysaccharide significantly enhanced the EBV lytic cycle in a concentration of 30 μg/mL, which indicated its potential usage as an antiviral drug.	(Guo et al., 2014)
Iota‐carrageenan	Human rhinovirus, human coronavirus, and influenza A virus	In vivo (clinical trials)	Use of carrageenan nasal spray in patients suffering from viral common cold decreased the duration and relapses of symptoms of disease and improved the viral clearance.	(Koenighofer et al., 2014)
Milk oligosaccharides	Human rotavirus strains	In vitro (MA104 cells)	Milk oligosaccharides diminished the infectivity of human rotaviruses in vitro with confirmed antiviral effect.	(Laucirica et al., 2017)
Fructan from Chikuyo—Sekko–To	Herpes simplex virus type 2 HSV‐2 influenza A virus (H1N1)	In vitro (RAW264.7 cells); in vivo (mice)	The extracted polysaccharide had modulatory effects on nitric oxide production and also induction of several cytokine mRNA expression, including IL‐1β, IL‐6, IL‐10, and TNF‐α.	(Lee et al., 2012)
Fructan from Welsh onion (*Allium fistulosum* L.)	Influenza A virus (H1N1)	In vitro (MDCK cells); in vivo (mice)	The polysaccharide improved the level of neutralizing antibodies against infection by influenza A virus.	(Lee et al., 2012)
Oat fiber β‐glucan	Herpes simplex virus type 1	In vivo (mice)	Macrophages are partially responsible for the antiviral effects of oat β‐glucan.	(Murphy et al., 2008)
Fucoidan from brown algae Kjellmaniella crassifolia	Influenza A virus	In vitro (MDCK cells) and in vivo (mice)	Fucoidan possessed antiviral activities both in vitro and in vivo, and could block the viral invasion and release through the cellular EGFR pathway.	(W. Wang et al., 2017)
Human milk oligosaccharides	Rotavirus	In vitro (MA‐104 cells); in vivo (piglets)	Human milk oligosaccharides inhibited the rotavirus infectivity in vitro and also decreased NSP4 replication through the acute rotavirus infection in vivo.	(Hester et al., 2013)
Milk oligosaccharides	Human rotavirus strains	In vitro (MA104 cells)	Milk oligosaccharides decreased the infectivity in MA104 cells by human rotaviruses. So, the addition of the oligosaccharides may be beneficial in infant formula.	(Laucirica et al., 2017)
κ‐carrageenan oligosaccharides	Influenza A virus	In vitro (MDCK cells) and in vivo (mice)	Carrageenan oligosaccharides and their sulfated derivatives had an ideal inhibitory impact on replication of influenza A virus both in vitro and in vivo.	(Wang et al., 2017)

Chitosan is a linear, alkaline, positive‐charged polysaccharide made by repeated glucosamine and N‐acetylglucosamine units derived from the fungal cell walls or the shells of shrimps and crustacean. Chitosan and its derivatives have shown a good inhibitory effect against different viruses that open new windows for further coronavirus research. The cationic derivative of chitosan (N‐(2‐hydroxypropyl)−3‐trimethylammonium chitosan chloride (HTCC)) exhibited an inhibitory impact on various human coronaviruses, such as HCoV‐OC43, HCoV‐229E, HCoV‐HKU1, and HCoV‐NL63. On the other hand, a hydrophobic derivative of chitosan (HTCC) has shown a considerable inhibitory effect against HCoV‐NL63, which reveals that HTCC‐based derivatives of chitosan are potent inhibitors against pathogenic human coronaviruses (Milewska et al., 2016). It has been shown that 10–100 μg/mL of N‐palmitoyl‐N‐monomethyl‐N,N‐dimethyl‐N,N,N‐trimethyl‐6‐O‐glycolchitosan can decrease the rate of Covid‐19 infection in human lung cells by 3–4 log values. This finding might be due to electrostatic binding to the coronavirus, which can inhibit the viral entry into the cells (Pyrć et al., 2020). Chitosan can promote the antigen‐specific immune responses triggered by the RSV via elevating the induction of lung T cells, regulatory T cells, and neutralization of antibodies as well as stimulation of cytotoxic and proliferative function of splenic leukocytes (Ghendon et al., 2009). The effect of chitosan as a stimulator of cell‐mediated and humoral immune responses with a proven safety record in animal models and human volunteers led to introduction of this polysaccharide as a promising adjuvant candidate for enhancing vaccine efficacy against the novel coronavirus (Chen et al., 2020). The potential of chitosan as an antiviral agent and a vaccine adjuvant makes it a critical molecule in formulating both antiviral agents and vaccines. However, various physical and chemical procedures in the preparation of chitosan from chitin have been developed, which significantly affect the final quality. The variation in source and preparation process of chitosan has resulted in production of a broad range of chitosan polymers with different physicochemical effects that may lead to contradictory reports regarding their performance (Jaber et al., 2021).

Carrageenan as sulfated linear polysaccharides extracted from red algae and made of repeating disaccharide units of β‐D‐galactopyranose, α‐galactopyranose, and 3,6‐anhydro‐α‐ galactopyranose. Carrageenan antiviral effect is due to its primary prevention of the entry or the binding of virions into the host cells (Chen et al., 2020). In a recent study, some marine sulfated polysaccharides were evaluated regarding their inhibitory activity against coronavirus, and among them fucoidan, and iota‐carrageenan, and sea cucumber sulfated polysaccharide exhibited a significant antiviral effect at concentrations of 3.90–500 μg/mL. The polysaccharides could be applied to prevent and treat COVID‐19 (Song et al., 2020).

Alginate, a linear and acidic polysaccharide derived from brown algae, is composed of α‐L‐guluronic acid and β‐D‐mannuronic acid. Alginate is a natural polysaccharide with approved immune activator property that has recently been proposed as a therapeutic regime for COVID‐19 patients. Alginate could successfully suppress the cytokine storm initiated by the coronavirus (El‐Sekaily et al., 2020). Furthermore, a sulfated derivative of alginate (polyguluronate sulfate) can appropriately inactivate the hepatitis B virus by Raf/MEK/ERK and NF‐κB signaling pathways to trigger the interferon system (Wu et al., 2016).

Fucoidan is a sulfated and fucose‐enriched polysaccharide obtained from different sources of brown algae mainly composed of sulfate groups, L‐fucose, and a small ratio of D‐mannose, D‐xylose, D‐glucuronic acid, and D‐galactose (Chen et al., 2020). Sulfated polysaccharides such as fucoidan can inhibit or interfere with the activation and expression of epidermal growth factor receptor (EGFR) as the main pathway causing pulmonary fibrosis. Prevention of the EGFR signaling pathway can inhibit the excessive fibrotic responses to respiratory viral infections such as SARS‐CoV (Venkataraman & Frieman, 2017).

*Astragalus* polysaccharide extracted from a Chinese herbal medicine called *Astragalus membranaceus* is a bioactive component consisting of mannose, D‐galactose, and D‐glucose. *Astragalus* polysaccharide at a concentration of 30 μg/mL, which is nontoxic, can significantly prevent the expressions of Zta and Rta viral proteins in the gammaherpesvirus 4 lytic cycle and possess antiviral activity (Guo et al., 2014).

β‐Glucan can be derived from different sources, such as algae, fungi, yeast, and plant, and has been well documented regarding immunostimulatory properties. The activation of β‐glucan–specific receptors on neutrophils, macrophages, and NK cells is responsible for the enhanced activities of the innate and specific immune system (Murphy et al., 2008). Both in vitro and in vivo studies revealed that the immunostimulatory effect of β‐glucan depends on the molecular weight, structure, and the number of branches (Khan et al., 2018). Lentinan in *Lentinus edodes* as a widely edible mushroom composed of a β‐glucan backbone and glucosyl‐branching units ended by galactosyl or mannosyl residues. Typically, mushrooms are known as an essential source of polysaccharides with immunomodulatory effects (Suwannarach et al., 2020). Lentinan, an extensively investigated polysaccharide with approved immunomodulatory processes, has been widely applied as a dietary supplement or alternative medicine (Chen et al., 2020; Zhang et al., 2011). Lentinan can downregulate the expression of IL‐2, IL‐11, and TNF‐α and upregulate the expression of IFN‐γ and IFN‐1 after exposure to the hematopoietic necrosis virus, which could be attributed to its ability in regulating the specific immunity and innate immune responses (Ren et al., 2018). The polysaccharides may react directly via preventing the adsorption and uptake of the viruses into the cells or inhibiting the viral enzymes (Khan et al., 2018).

Fructans are water‐soluble compounds naturally found in flowering plants, e.g., tomato, onion, garlic, rye, banana, chicory, barley, dragon fruit, asparagus, and honey. Both long‐chain fructans (inulin: DP > 10) and short‐chain ones (fructooligosaccharides: DP < 10) are well‐known prebiotic compounds exerting overall health effects by stimulation of the proliferation and growth of beneficial intestinal bacteria (Dobrange et al., 2019). Their immunomodulatory function is dependent on Toll‐like receptors (TLR 2, 4, 5, 7, 8). After binding to the receptors, some signaling pathways might be triggered, which is dependent on the nuclear factor NF‐κB, peptidoglycan recognition protein 3 (PGlyRP3), and peroxisome proliferator‐activated receptors (Peshev & Van den Ende, 2014). Fructans isolated from Chikuyo–Sekko–To, a traditional Japanese herbal medicine, have shown antiviral effects against herpes simplex virus type 2 in vivo and in vitro via enhancement of the production of nitric oxide as a viral replication inhibitor as well as other immunostimulatory factors such as IL‐1β, IL‐10, IL‐6, TNF, and IFN (Lee et al., 2012). Other investigations on both long‐ and short‐chain fructans from fresh and aged garlic have demonstrated the capacity to activate the macrophages and then phagocytosis in combination with the release of nitric oxide (Dobrange et al., 2019).

Oligosaccharides as low‐molecular‐weight carbohydrates between mono‐ and polysaccharides can be obtained from natural sources or chemically synthesized from disaccharides as well as the hydrolyzation of polysaccharides. The special properties of oligosaccharides including low‐viscosity and high‐solvency at neutral pH, nonallergenic, nontoxic, disease‐preventing, and health‐promoting characteristics improved their potential to be used as pharmaceuticals and drugs (Ji et al., 2011). Some important bioactive oligosaccharides include xylooligosaccharides, fructooligosaccharides, β‐glucan oligosaccharides, galactooligosaccharides, mannan oligosaccharides, pectic oligosaccharides, iso‐maltooligosaccharides, arabinooligosaccharides, chitosan oligosaccharides, algae‐derived marine oligosaccharides, and human milk oligosaccharides, etc. These oligosaccharides are naturally present in milk, fruits, honey, lentils, sugarcane juice, and vegetables (Bhatia et al., 2019). Subsequently, some potentially known antiviral oligosaccharides are discussed.

Xylooligosaccharides have a critical role in the development of the oligosaccharides market. Its cost is quite variable, which could be due to the diverse purity. Different hemicellulosic sources such as Bengal gram husk, corn stalks, corn cob, wheat bran and straw, pigeon pea stalks, barley hulls, sugarcane bagasse, green coconut husks, and algal sources have been identified for the production of xylooligosaccharides. The structure is composed of 2–20 units of xylose joint by β‐1,4‐xylosidic linkage (Belorkar & Gupta, 2016; Bhatia et al., 2019). The main bioactive roles related to the xylooligosaccharides have been reported as antioxidant, prebiotic, gelling agent, treatment of diabetes, antitumor, and antiviral agent (Suwannarach et al., 2020). Sulfated xylooligosaccharides extracted from Red seaweed *Nothogenia fastigiata* were found to show antiviral activity against types 1 and 2 of herpes simplex virus (Gupta et al., 2016).

The antiviral effect of chitosan oligosaccharides involves the activity of macrophages, which can enhance the production of active oxygen species and subsequent viral destruction. Another suggested mechanism is related to the interactions between blood leucocytes and viral coat receptors. The positive relationship between the antiviral activity of chitosan oligosaccharides and their positively charged groups has also been proven (Ji et al., 2011).

Carrageenan oligosaccharide with exclusive properties, such as abundance, nontoxicity, and biodegradability, can be used as biocompatible reductants for green synthesis of gold nanoparticles (Chen et al., 2019). The oligosaccharide capped gold nanoparticles can be loaded by S or N protein obtained from coronavirus and be applied for vaccine designation (Chen et al., 2020).

Human milk oligosaccharides found in breast milk have a unique structural variety composed of five monosaccharides, including D‐glucose, D‐galactose, *N*‐acetylglucosamine, L‐fucose, and *N*‐acetylneuraminic acid bonded by glycosidic linkages. These oligosaccharides can provide an indirect nutritional value to the infant via promoting the growth of beneficial intestinal microflora and subsequently the generation of short‐chain fatty acids. They can also directly modulate immune responses and decrease selectively the binding of pathogenic bacteria and viruses to epithelial cells (Walsh et al., 2020). Further, in vivo studies by Hester et al. (2013) exhibited that both neutral and acidic fractions significantly diminished the infectivity of rotavirus. Laucirica et al. (2017) also revealed the strain‐specific effect of human milk oligosaccharides on infectivity reduction of two dominant rotavirus strains in MA104 cells (monkey kidney epithelial cells). So, it is plausible to speculate that human milk oligosaccharides may act as antiviral agents against SARS‐CoV‐2. The potential of these oligosaccharides to behave as soluble decoy receptors for the coronavirus could be of great interest in further investigations (Walsh et al., 2020).

### 3.7 Bioactive peptides and bioactive protein fractions

Bioactive peptides (consisting of 3–20 amino acid residues) and proteins (generally consisting of more than 20 amino acids) are functional food ingredients that offer several advantageous bioactivities, such as antioxidant, antihypertensive, antimicrobial, antidiabetic, immunomodulatory, antiobesity, cytomodulatory, and antithrombotic properties (Goudarzi & Madadlou, 2013; Sarmadi & Ismail, 2010). Relying on the fractionated sequence, bioactive peptides are classified into the ingredients effective in human nervous, gastrointestinal, cardiovascular, and immune systems (Sánchez & Vázquez, 2017). Bioactive peptides remain inactive as long as their relevant sequences are entrapped by long chains of proteins (Goudarzi et al., 2012). The release of bioactive peptide sequences from the protein matrix is facilitated through the fermentation process, enzymatic hydrolysis, and gastrointestinal digestion of protein‐containing foods. Most of the bioactive peptides and protein fractions are originated from milk and dairy products, meat, egg, fish, poultry, algae, nuts, and seeds (Mohanty et al., 2016).

The side effects of synthetic antiviral drugs together with high mutability of coronaviruses have encouraged scientists to use natural antivirals as a sustainable long‐term strategy for remediation of coronavirus infections (Real et al., 2004). In this regard, the antimicrobial protein fractions and bioactive compounds are developed as the body initial shield against various pathogens mainly through their immune‐boosting functions. Impediment in virus reproduction capacity, interruption in the attachment of virus‐cell membrane surface by blocking cell receptors, and direct destruction of virus integrity are pointed out as further antiviral mechanisms of bioactive peptides and bioactive protein fractions (da Mata et al., 2017).

The protein griffithsin, which is a lectin of 121 amino acids derived from the red algae *Griffithsia*, has shown potential to prevent and treat a wide range of viral infections (Decker et al., 2020). Griffithsin is a homodimeric complex where each monomer harbors three carbohydrate‐binding domains (Lee, 2019). The ability of griffithsin to bind to oligo‐saccharides makes it a promising candidate to block spike protein, the highly glycosylated surface protein of coronaviruses, which in turn inhibits the virus infusion into the host cell (O'Keefe et al., 2010). Ziółkowska et al. (2006) reported that all three crystal forms of griffithsin including un‐liganded trigonal SeMet, un‐liganded orthorhombic, and N‐glucosamine complex could significantly inhibit SARS‐CoV even at nanomolar concentrations, either by interfering with virus replication or by their CPEs on virus integrity. Similarly, O'Keefe et al. (2010) studied the in vivo and in vitro antiviral effects of griffithsin protein on SARS‐CoV (O'Keefe et al., 2010). They disclosed that the main antiviral mechanism of griffithsin is the interaction between griffithsin active sites and SARS‐CoV spike glycoproteins. They also indicated the antiviral effects of griffithsin against other coronaviruses infecting mammals and birds. The in vivo experiment on mice confirmed the Griffithsin‐treated SARS‐CoV–infected cases experienced positive responses and lower mortality rates. In line with these results, Barton et al. (2014) reported potent antiviral activity for griffithsin against various viral infections including SARS‐CoV diseases.

Lactoferrin, owing to its broad‐spectrum antiviral activity, is another protein that is attracting broad research interest as a natural weapon for fighting epidemic and pandemic viral infections. Lactoferrin is a glycosylated protein consisting of a single chain of approximately 700 amino acids. It is folded into two symmetrical lobes (N‐terminal and C‐terminal), each of which possesses an iron‐binding site (Miotto et al., 2021). Lactoferrin is present in mucosal secretions such as saliva, tears, nasal secretions, gastrointestinal fluids, urine, seminal and vaginal fluids, and most of all in milk (González‐Chávez et al., 2009). Lang et al. (2011) reported that lactoferrin inhibited SRAS pseudovirus cell entry through blocking cell‐surface heparan sulfate proteoglycans (HSPGs). Widely distributed on the mammalian cell membranes, HSPGs are thought as the initial docking site for a range of viruses invading human cells including HIV type 1, hepatitis B and C viruses, human papillomavirus, herpes virus, foot, and mouth disease virus, dengue virus, and most importantly SARS‐COV (Lang et al., 2011). In fact, before binding to ACE2 receptor and subsequent internalization into host cells, SARS‐COV most probably attaches to HSPGs via spike protein, although with lower affinity compared with attachment to ACE2. It was deduced that the positively charged N‐terminal glycosaminoglycan‐binding domain of lactoferrin binds to negatively charged HSPGs via electrostatic attraction, which in turn interferes the virus initial attachment to host cells (Lang et al., 2011). The findings of this study, along with the well‐known antiviral activities of lactoferrin, have recently inspired several researchers into exploring the possible inhibitory effect of lactoferrin on SARS‐COV‐2. In an in vitro study, de Carvalho et al. (2020) reported that bovine lactoferrin potently interfered with SARS‐CoV‐2 replication in a dose‐dependent manner (0.2–1.0 mg/mL). In fact, based on RNA level, SARS‐CoV‐2 yields were reduced by approximately 84.6% and 68.6% in African green monkey kidney epithelial cells and in adenocarcinomic human alveolar basal epithelial cells, respectively, at 1 mg/mL lactoferrin concentration. Later, Hu et al. (2021) showed that both bovine lactoferrin and human lactoferrin inhibited SARS‐COV‐2 replication in multiple cell lines, implying the cell type independency of antiviral effect of lactoferrin and that they may provide protection against the coronavirus infection in different organs. It was also found that lactoferrin exerted its inhibitory effect on viral replication through blocking cell‐surface HSPG receptors at the viral attachment stage, whereas no noticeable antiviral effect was observed when lactoferrin was included during viral entry or post‐viral entry stages (Hu et al., 2021). Note that the combined use of lactoferrin with the FDA‐approved SARS‐CoV‐2 antiviral remdesivir showed synergistic antiviral activity in cell culture (Hu et al., 2021). Similarly, Cegolon et al. (2021) reported higher in vitro virucidal activity of hypothiocyanite against SARS‐CoV‐2 when it was applied in combination with lactoferrin.

The preventive and soothing effects of lactoferrin on SARS‐COV‐2 infection have also been demonstrated in clinical trials. Serrano et al. (2020) reported that the COVID‐19 patients with typical symptoms who were orally administrated with a liposomal bovine lactoferrin nutritional syrup food supplement recovered completely in the first 4–5 days of the trial. Moreover, the same treatment but at lower doses prevented the infection of healthy individuals who were directly in contact with patients. A similar study found that oral and intranasal treatment of mild‐to‐moderate and asymptomatic COVID‐19 patients with liposomal bovine lactoferrin significantly reduced the mean time length of rRT‐PCR SARS‐COV‐2 RNA‐negative conversion in comparison with the standard‐of‐care (SOC)‐treated and nontreated patients (Campione et al., 2020). Lactoferrin supplementation also caused significant decreases of inflammatory biomarkers, namely, ferritin, IL‐6, and D‐dimer, which in turn resulted in faster clinical symptoms recovery of the patients (Campione et al., 2020). The downregulating impact of lactoferrin on inflammatory blood parameters of SARS‐COV‐2–infected individuals was also reported by Serrano et al. (2020). The anti‐inflammatory of lactoferrin is believed to be due to its capability to chelate iron and thus restore iron homeostasis, which is disturbed by viral infection (Campione et al., 2020).

There is a dearth of experimental data on the potential of food protein‐derived bioactive peptides for preventing or treating the epidemic infections caused by coronaviruses MERS‐CoV, SARS‐COV, and SARS‐COV‐2. However, food proteins are thought as potential sources for mining cathepsin L‐inhibitory peptides to combat such diseases (Madadlou, 2020). Cathepsin L is a host cell protease that plays a critical role in endocytosis of SARS‐COV and SARS‐COV‐2 by cleaving their protein S (Salaris et al., 2021). Therefore, inhibition of cathepsin L protease could be a promising strategy to block or substantially reduce the entry of the viruses into the host cells. The peptides with cathepsin L‐inhibitory activity have already been identified in rice (Hellinger & Gruber, 2019) and pineapple stem (Polya, 2003). Furthermore, milk proteins and in particular lactoferrin, which itself has shown cathepsin L‐inhibitory activity (Sano et al., 2005), hold great promise for generating such antiviral peptides (Madadlou, 2020). Bioactive peptides of food origins might also have implications on endocytosis and pulmonary function of SARS‐CoV‐2 in affected patients through influencing the renin–angiotensin system (Madadlou, 2020). The presumed mechanisms of action of such peptides have been outlined elsewhere (Goudarzi et al., 2020). Notwithstanding, whether food‐derived bioactive peptides can prevent and/or treat coronavirus infections remains to be explored.

### 3.8 Essential oils

Ethereal or volatile oils are aromatic compounds (> 17,000) produced as secondary metabolites in many parts (fruits, buds, flowers, leaves, stems, twigs, bark, roots, seeds, and wood) of numerous higher plants, namely, *angiospermic families Lamiaceae*, *Asteraceae*, *Zingiberaceae*, *Myrtaceae*, and *Rutaceae*, called by the term “essential oils” (EOs; Burt, 2004; Regnault‐Roger et al., 2012). Usually, antimicrobial effects of EOs are affected by both concentrations of EO and their molecular structure (El Hadi et al., 2013). In fact, EOs consist of more than 100 bioactive components, including hydrocarbons such as terpenes and sesquiterpenes, and oxygenated compounds like phenols, among which two or three components have usually a major share of EO constituents varied from 20% to 70%, determining their biological activities and characteristics (Islam et al., 2016; Pandit et al., 2016). Although some components of EOs (mostly terpenes, phenolics, and aldehydes) are considered as key agents against viruses, molds, microbial toxins synthesis, bacteria, and insects (Shahidi & Hossain, 2018; Swamy et al., 2016), it is believed that the biological activities of EOs are frequently attributed to a combined effect of their constituents (Bekut et al., 2018; Tariq et al., 2019). As an example, Schnitzler et al. (2008) claimed that the application of original EOs was biologically better than individual components, as different compounds present in lemon balm EO influenced different phases in the life cycle of type 1 and 2 herpes simplex viruses (HSV‐1 and HSV‐2) via different mechanisms. On the other hand, Astani et al. (2010) reported that the single monoterpenes and EOs from eucalyptus, thyme, and tea tree were able to inhibit HSV‐1 in vitro by more than 80% and 96%, respectively, indicating both significance of individual components and the synergistic effect of EO components as antiviral agents.

*Ex vivo*, in vivo, and in vitro studies suggested that antiviral activity of EOs can be indirect, through promoting the immunity of human or synergistic effects on conventional drugs, or maybe a direct effect at which EO components target the outer structures of viruses (primarily viral envelop), disabling the attachment ability of virus when it is close to the target cell (Astani et al., 2011; Bekut et al., 2018; Gilling et al., 2014). Moreover, based on time‐of‐addition studies dealing with antiviral activities of EOs extracted from ginger, hyssop, anise, thyme, sandalwood, chamomile (Koch et al., 2008), *Santolina insularis* (De Logu et al., 2000), and data presented in Table 4, it is claimed that the activity of the EOs is mostly noticeable when cells or viruses are treated before the challenge.

**TABLE 4:** Effect of different plant‐derived essential oils (EOs) on selected viruses, especially coronaviruses

			EO properties				
Virus	Trial type	Treatment	Major component(s)	IC_50_	SI	Effect(s)	References
Human coronavirus strain NL63, hRv, H1N1 influenza, and hMpv	Clinical examination as a double‐blind randomized controlled trial	Capsules containing olive oil and a blend of EOs from (1) Thyme (*Coridothymus capitatus* (L.)), (2) Cretan dittany (*Origanum dictamnus* L.), and (3) Sage (*Salvia fruticosa* Mill.)	(1) Carvacrol (2) Carvacrol and *p*‐cymene (3) Eucalyptol	–	–	‐ Reduction in upper respiratory tract infection only within the intervention group. ‐ Relatively high cessation of symptoms in virus‐positive patients.	(Duijker et al., 2015)
Influenza A virus strains (H1N1 and H3N2), influenza B, hRv14 and, HRSV	In vitro (MDCK and HeLa cells)	CAPeo containing olive oil and a blend of EOs from (1) Thyme (*Coridothymus capitatus* (L.)), (2) Cretan dittany (*Origanum dictamnus* L.), and (3) Sage (*Salvia fruticosa* Mill.)	(1) Carvacrol (2) Carvacrol and *p*‐cymene (3) Eucalyptol	–	–	‐ Low cytotoxicity of CAPeo in vitro. ‐ Highly inhibition of H1N1 strains by CAPeo, but inactive for H3N2. ‐ CAPeo targeted H1N1 or hRv14 after entry in host cells and affected HRSV before the entry. ‐ A nucleoprotein in influenza A was target site for antiviral agent.	(Tseliou et al., 2019)
Two serotypes of coronavirus avian IBV	In vitro (Vero E6 cells and embryonating eggs) and in vivo (chickens)	QR448(a) containing a mixture of botanical oleoresins and EOs	–	–	–	‐ Inhibition of IBV in both trials. ‐ QR448(a) was the most effective 2 h prior to encounter with IBV. ‐ Direct virucidal effect of QR448(a) on IBV.	(Jackwood et al., 2010)
Coronavirus IBV	In vitro (Vero cells from African green monkey kidney), in ovo (chicken embryos), and in vivo (chickens)	Solution of *Houttuynia cordata*. (HC), mostly containing EOs	Methyl‐nonyl‐ketone (2‐undecanone)	–	–	‐ A protection rate of 50%–90% against IBV infection by treatment before challenge in vitro and in vivo. ‐ Direct virucidal effect of HC on IBV. ‐ Weak protection for virus‐infected subjects.	(Yin et al., 2011)
SARS‐CoV and HSV‐1	In vitro (Vero cells)	EOs: (1) *Laurus nobilis* (2) Juniperus oxycedrus ssp. oxycedrus	(1) β‐Ocimene, 1,8‐Cineole, α‐Pinene, and β‐Pinene (2) α‐Pinene and β‐myrcene	(1) 120 μg/mL (2) 200 μg/mL	(1) 4.16 (2) 5	(1) Strong antiviral activity of EO against SARS‐CoV. (2) Antiviral activity of EO against HSV‐1.	(Loizzo et al., 2008)
SARS‐CoV	In vitro (Vero E6 cell)	Ethyl acetate extracts from heartwood of Chamaecyparis obtusa var. formosana and Juniperus formosana	**(I)** Terpenoids: (1) Ferruginol; (2) [8β‐hydroxyabieta‐9(11),13‐dien‐12‐one]; (3) 7β‐ Hydroxydeoxy‐cryptojaponol); (4) 3β,12‐Diacetoxyabieta‐6,8,11,13‐tetraene; (5) Betulonic acid **(II)** Lignoids: (6) Savinin	(1) 1.39 (2) 1.47 (3) 1.15 (4) 1.57 (5) 0.63 (6) 1.13	(1) 58 (2) > 510 (3) 111 (4) 193 (5) 180 (6) > 667	‐ Strong antiviral activity of abietane‐ and lupine‐type terpenoids, and lignoids against SARS‐CoV.	(Wen et al., 2007)
HSV‐1, DENV‐2, and JUNV	In vitro (Vero (cells from African green monkey kidney)	EOs: (1) Romerillo (Heterothalamus alienus) (2) Salvia blanca (Buddleja cordobensis)	(1) β‐Pinene, sphatulenol, and Germacrene D (2) Caryophylene oxide, β‐caryophylene, and α‐copaene	(1) HSV‐1: 148.4, DENV‐2: 122.3 and JUNV: 44.2 (2) HSV‐1: 54.1, DENV‐2: 86.4 and JUNV: 39.0	(1) HSV‐1: 0.99, DENV‐2: 1.21 and JUNV: 3.34 (2) HSV‐1: 2.91, DENV‐2: 1.82 and JUNV: 4.03	‐ Antiviral agents directly interacted with virions. ‐ JUNV was the most inhibited virus. ‐ EO‐inactivated virions maintained their ability to bind to the host cell.	(Duschatzky et al., 2005)
ACVR‐HSV‐1, HSV‐1, HRSV, BoHV types ‐1, ‐2, and ‐5, BVDV, and human RV	In vitro (MDBK cells, MA104 cells, and HEp‐2 cells) EO addition: (I) Before and after viral inoculation. (II) Only after viral inoculation	(1) EO of Mexican oregano (*Lippia graveolens*) rich in carvacrol (2) Single carvacrol	Carvacrol	(1) (I) ACVR‐HSV‐1: 55.9 (I) HSV‐1: 99.6 (I) HRSV: 68 (II) BoHV‐2: 58.4 (II) BVDV: 78 (2) (II) RV: 27.9	(1) ACVR‐HSV‐1: 13.1 HSV‐1: 7.4 HRSV: 10.8 BoHV‐2: 9.7 BVDV: 7.2 (2) RV: 33	‐ Unlike the single component, EO inhibited different human and animal viruses in vitro, likely due to synergistic effect of components. ‐ EO inhibited viruses before and after inoculation, whereas carvacrol was effective after virus inoculation.	(Pilau et al., 2011)
CV‐B4	In vitro (HEp‐2 cells)	EO of wild *Daucus virgatus* (Poir.) Maire	Methyl eugenol and β‐bisabolene	–	–	‐ Moderate antimicrobial activities and weak DPPH radical scavenging ability. ‐ No antiviral activity.	(Snene et al., 2017)
CV‐B4	In vitro (HEp‐2 cells)	EO of *Osmunda regalis*	Diterpenoid hexahydrofarnesyl acetone, 2,4‐di‐tbutylphenol and phytol	2.24	789.66	‐ Strong antiviral activity against CV‐B4	(Bouazzi et al., 2018)
HSV‐1	In vitro (Vero cells from African green monkey kidney)	EOs: (1) *Zataria multiflora* Boiss (2) *Rosmarinus officinalis* (3) *Artemisia kermanensis* (4) *Satureja hotensis L* (5) *Eucalyptus caesia*	(1) Thymol and carvacrol (2) α‐Pinene and verbenon (3) α‐Thujone, camphor, and β‐thujone (4) Carvacrol, γ‐terpinene (5) 1,8‐Cineol, p‐cymene, γ‐terpinene	(1) 30 (2) 60 (3) 40 (4) 80 (5) 60	(1) 55.44 (2) 46.12 (3) 66.37 (4) 32.16 (5) 38.81	‐ Significant inhibition of HSV‐1. ‐ Increased concentration of EOs inhibited virus plaque formation.	(Gavanji et al., 2015)
MNV strain S7‐PP3	In vitro (RAW 264.7 cell line monolayer)	Oregano EO‐rich in carvacrol and purified carvacrol	Carvacrol	–	–	‐ Both ingredients appeared to act directly the viral capsid and subsequently the RNA. ‐ Virus adsorption did not appear to be affected by ingredients. ‐ Carvacrol was more effective than oregano oil with very higher concentrations at all exposure time intervals.	(Gilling et al., 2014)
HSV‐1	In vitro (RC‐37 cells from African green monkey kidney)	(I) EO of star anise rich in trans‐anethole (II) Individual phenylpropanoids and sesquiterpenes	(1) Trans‐anethole (2) Eugenol (3) β‐Eudesmo (4) Farnesol (5) β‐Caryophyllene (6) β‐Caryophyllene oxide	(I) 1 (1) 20 (2) 35 (3) 6 (4) 3.5 (5) 0.25 (6) 0.7	(I) 160 (1) 5 (2) 2.4 (3) 5.8 (4) 11.4 (5) 140 (6) 25.7	‐ (I) and (5) were the most potent antivirals against HSV‐1. ‐ Ingredients directly inactivated HSV‐1, affecting the virion envelope structures or masking the structures necessary for adsorption or entry into host cells. ‐ Antiviral activity occurred through different mechanisms.	(Astani et al., 2011)

SI: selectivity index (CC_50_/IC_50_); IC_50_: 50% inhibitory concentration (μg/mL); CC_50_: 50% cytotoxic concentration (μg/mL); MDBK: Mardin–Darby bovine kidney; HEp‐2: human epithelial cell line type 2; HSV‐1: herpes simplex virus type 1; ACVR‐HSV‐1: acyclovir‐resistant herpes simplex virus type 1; hMpv: human metapneumovirus; hRv: human rhinovirus; IBV: infectious bronchitis virus; HRSV: human respiratory syncytial virus; BoHV‐1, ‐2, and ‐5: bovine herpesvirus types 1, 2, and 5; BVDV: bovine viral diarrhea virus; SARS‐CoV: severe acute respiratory syndrome‐associated coronavirus; DENV‐2: dengue virus type 2; JUNV: Junin virus; CV‐B4: coxsackievirus B4; MNV: murine norovirus.

Some studies investigating the antiviral activity of various plant‐derived EOs, especially against coronaviruses, have been listed in Table 4. In 1995, 100 medicinal plants grown in British Colombia (Canada) were introduced to have antiviral effects, 12 of which had significant antiviral activity against parainfluenza virus type 3 (PI‐3), coronaviruses, RSV, rotavirus, and HSV‐1 (McCutcheon et al., 1995). In 2005, antiviral influences of many common Chinese herbal medicines (> 200) on SARS‐CoV were evaluated, four of which exhibited strong activities (Li et al., 2005).

Loizzo et al. (2008) tried to inhibit SARS‐associated coronavirus (SARS‐CoV) by EOs from *Laurus nobilis* leaves. Despite a strong antiviral activity with IC_50_ value of 120 μg/mL, they suggested ignoring the EOs for therapeutic application due to its relatively low selectivity index (SI, 4.6), as it is generally accepted that a drug with SI value more than 10 has a good safety profile (Tamargo et al., 2015). Jackwood et al. (2010) examined the activity of a synergistic mixture of botanical oleoresins and EOs, designated QR448(a) from Quigley Pharma, Inc. (Pennsylvania, USA), on a coronavirus named avian infectious bronchitis virus (IBV) in vivo and in vitro. The anti‐coronaviral agent was successful to inhibit different serotypes of IBV in both procedures, as viral load reduced in laboratory host systems, and a significant decrease recorded for both viral RNA load in the trachea and the severity of clinical symptoms and lesions in chickens. Moreover, in vivo study showed that treatment of the birds with QR448(a) was most effective 2 h before encountering coronavirus IBV (indicating the virucidal effect of QR448(a) before virus attachment and entry), and protected chickens from disease clinical signs for up to 4 days and diminished IBV transmission for up to 14 days. In a similar study (Yin et al., 2011), a commercial *Houttuynia cordata* (HC) solution, mostly containing EOs (Table 4) used to inhibit the coronavirus IBV infection in vitro, in vitro (in the egg), and in vivo. They confirmed that IBV, similar to other coronaviruses, may induce the apoptosis of cells, which can be inhibited due to the direct virucidal effect of HC on IBV. HC showed a protection rate of > 90% against IBV infection in kidney cells of specific pathogen‐free (SPF) chicken embryos and Vero cells, whereas its inhibition rate in SPF chickens was more than 50%. However, HC did not protect the cells infected with IBV and had no full protection for virus‐infected chickens. Therefore, they recommended applying HC along with other anti‐IBV drugs or vaccines to have a better outcome.

Duijker et al. (2015) used a randomized placebo‐controlled, double‐blind, parallel‐group design to study the effectiveness of EO (15 mL of EO per L of olive oil) from Cretan aromatic plants on upper respiratory tract infections (induced by human parainfluenza virus 1–4 (hPiv) RNA, HKUI human coronavirus (hCov) RNA, etc.) in patients aged ≥ 18 years (placebo and treated groups with 51 and 54 persons, respectively) as they treated for 7 days with two 0.5 mL capsules daily (Table 4). They observed no significant differences in symptom severity or duration between the groups. However, using an analysis restricted within each group, they observed a significant reduction for both proportion and number of patients with constantly increased CRP (C‐reactive protein) level—a sign of infection improvement—only within the intervention group. Moreover, on the sixth day of treatment, 91% of the treated population versus 70% of the placebo participants had no symptoms of disease when the analysis was restricted to virus‐positive patients. Finally, they called for additional studies with a greater number of patients and a higher dose of EOs.

Therefore, EOs are promising candidates for the treatment of infections induced by coronaviruses like new coronavirus (SARS‐CoV‐2), and further research is required to evaluate the single and combined effects of constituents of many potential plant‐derived EOs, as well as their safety and recommended daily intake to be used alone, together or in combination with other preventive or therapeutic drugs because antiviral effects could be significantly enhanced using various components acting via different mechanisms.

### 3.9 Probiotics and their metabolites

Probiotics are naturally occurring alive microorganisms possessing innumerable health‐supporting attributes, acting as immune‐modulators, allergen alleviators, mental health promoters, gastrointestinal regulators, antihypertensive factors, and antimicrobials (Moghaddam et al., 2018; Sarlak et al., 2017). The superhealthy natural food products containing probiotic lactic acid bacteria (LAB) strains consist of fermented dairy products (e.g., yogurt, kefir, and cheeses), Tempe, Natto, Kombucha, Kimichi, Miso, etc. It has been stated that the market of probiotic‐enriched food products and supplements is progressively growing in the era of a pandemic due to their potential immune‐boosting features. As proven in clinical studies, probiotics can exert antiviral activities either due to the formation of virus‐probiotic interconnections or reinforcement of body immune system through stimulating specific immune‐signaling responses to physiological feedbacks (Lehtoranta et al., 2014). However, their meticulous mode of action at odds with the current SARS‐CoV‐2 virus is still ambiguous.

In the past few years, numerous in vivo and in vitro attempts have been engaged in animal models to inhibit or at least control various viral and bacterial infections. For instance, Gabryszewski et al. (2011) reported the protecting effects of activated (alive) and inactivated probiotic‐mediated (*Lb. plantarum* and *Lb. reurei*) priming of the respiratory mucosa against pneumonia viruses in mice. They pointed out that the activated bacilli‐treated respiratory mucosa eventuated in subsiding the expression of proinflammatory cytokines and eventually interrupting virus retrieval potency. Seo et al. (2010) investigated the antiviral effects of more than 300 strains of gut‐isolated bacteria upon transmissible gastroenteritis coronavirus in porcine and found the results of interest in two probiotic strains (*Lb. plantarum* and *Lb. salivarius*). The oral administration of these probiotic strains indicated the applicable antibiotic resistance in both in vivo and in vitro studies, candidate them as ideal alternatives instead of commonly used coronavirus‐treating antibiotics. In another study, the mucosal delivery of the *Lb. casei*‐mediated dendritic cell‐chaffing oral vaccine was scrutinized against porcine epidemic diarrhea coronavirus (PEDC; Wang et al., 2017). The in vivo oral administration of this probiotic‐mediated vaccine could effectively prompt the secretion of humoral, cellular, and mucosal immune responses against the studied coronavirus, introducing a propitious genetically fashioned vaccine.

Similarly, the antiviral effects of live and inactive (cell‐free supernatant) pig‐derived probiotic LAB strains on PEDC‐infected piglets were examined by Sirichokchatchawan et al. (2018). Among the studied LABs, the live active *Lb. plantarum* together with *Pediococcus pentoseceus* and all inactivated supernatants of LABs could significantly treat the PEDC‐infected piglets through stimulating the secretion of immune responses. In addition, the suitability of probiotic‐enriched feeds containing active bacteria of *Bacillus mesentericus*, *Streptococcus faecalis*, and *Clostridium butyricum* was investigated in the treatment of PEDC‐infected pregnant sows (Tsukahara et al., 2018). Rather than improvement in reproduction capability, the probiotic‐supplemented sows were more resistant upon PEDCs compared with the commonly fed ones, most likely due to their boosted immune system as a result of probiotic supplementation. In a unique study on using inactivated yogurt‐derived metabolites, it has been shown that the employed probiotic metabolites, especially those screened from *Lb. plantarum*, have antiviral activities against various RNA coronaviruses (Choi et al., 2009).

The clinical human approaches also confirmed the antiviral effects of probiotic bacteria against coronaviruses. In this regard, Olivares et al. (2007) designed a trial consisting of 30 human cases to diagnose the synergic influences of *Lb. fermentum* on efficiency of influenza vaccine. The results indicated that the oral administration of probiotic bacteria (dosage: 1 × 10^10^ CFU/day), 2 weeks earlier than and 2 weeks after vaccination, intensified the virus‐nullifying antibodies, and immune Th1 response, consequent to the improvement of vaccine efficiency compared with methylcellulose administration as placebo. In addition, the protective role of *Lb. reuteri* against upper respiratory tract symptoms and gastrointestinal disorders, and a decrease in diarrhea occurrence, has been proven through the in vivo trials on cases aged younger than six years (Agustina et al., 2012; Gutierrez‐Castrellon et al., 2014; Weizman et al., 2005).

Coadministration of probiotics strains and prebiotics (probiotic nourishments) has been demonstrated to be an effectual strategy in improving the immunogenicity of the influenza vaccine by affecting seroprotection and seroconversion amounts, more specifically in robust adults (Lei et al., 2017). The increase in the effectiveness of vaccines and body immunologic responses have been promoted by conjunct loading of probiotic bacteria and vitamin D supplements in functional foods (Aranow, 2011).

Taken as a whole, proper administration of probiotics via food and food supplements, in addition to all beneficial health‐promoting aspects, resulted in a safe and highly credited strategy to modulate the immune system and increase the vaccine effectiveness against various coronaviruses. However, further clinical and mechanistic studies should be addressed to understand their exact antiviral mechanisms in the treatment of coronavirus‐mediated respiratory disorders, in particular those infected by SARS‐CoV‐2.

## 4 CONCLUSION

The COVID‐19 pandemic has turned the public health upside down, with significant mortality and morbidity. No effective specific therapies are available to date. In addition, the mutagenic nature of coronaviruses has forced researchers to intend alternative approaches to treat coronavirus infections. Therapies with bioactives can be mainly divided into four groups: bioactive compounds inhibiting the virus enzymes, replication, and infection; bioactive compounds decreasing the ACE2 receptor activity; bioactive compounds suppressing the host inflammatory responses; and bioactive compounds boosting the human immune system against COVID‐19. Our review shows that several polyphenols, carotenoids, minerals, vitamins, oligosaccharides, bioactive peptides, and probiotics may inhibit COVID‐19 enzymes (3CL^pro^, PL^pro^, and RdRp), ACE2 receptor activity, and attenuated inflammatory responses. Moreover, these compounds can improve the immune system and, consequently, contribute to combat COVID‐19. In addition to the potent healing effect of these compounds, their low toxicity and low cost make them good antiviral candidates for use during such a pandemic. A strong immune system also plays a key role against coronavirus. Diets rich in bioactive compounds, fiber, vitamins, minerals, protein, minerals, oils, probiotics, etc., help fight virus infection and improve the immune system. However, the effects of these bioactive compounds against COVID‐19 are yet to be investigated further and confirmed in clinical trials and mechanistic studies.

## CONFLICT OF INTEREST

The authors confirm that they have no conflict of interest to declare for this publication.

## References

[fft2119-bib-0001] Achak, M. , Alaoui, S. B. , Chhiti, Y. , Alaoui, F. E. M. H. , Barka, N. , & Boumya, W. (2020). SARS‐CoV‐2 in hospital wastewater during outbreak of COVID‐19: A review on detection, survival and disinfection technologies. Science of the Total Environment, 761, 143192. 10.1016/j.scitotenv.2020.143192 PMC7585361 33153744

[fft2119-bib-0003] Agustina, R. , Kok, F. J. , Van De Rest, O. , Fahmida, U. , Firmansyah, A. , Lukito, W. , Feskens, E. J. M. , Van Den Heuvel, E. G. H. M. , Albers, R. , & Bovee‐Oudenhoven, I. M. J. (2012). Randomized trial of probiotics and calcium on diarrhea and respiratory tract infections in Indonesian children. Pediatrics, 129(5), e1155–e1164. 22492764 10.1542/peds.2011-1379

[fft2119-bib-0004] Alfaraj, S. H. , Al‐Tawfiq, J. A. , Assiri, A. Y. , Alzahrani, N. A. , Alanazi, A. A. , & Memish, Z. A. (2019). Clinical predictors of mortality of Middle East Respiratory Syndrome Coronavirus (MERS‐CoV) infection: A cohort study. Travel Medicine and Infectious Disease, 29, 48–50. 30872071 10.1016/j.tmaid.2019.03.004 PMC7110962

[fft2119-bib-0282] Alsaidi, S. , Cornejal, N. , Mahoney, O. , Melo, C. , Verma, N. , Bonnaire, T. , Chang, T. , O’Keefe, B. R. , Sailer, J. , Zydowsky, T. M. , Teleshova, N. , Romero, J. A. F. (2021). Griffithsin and Carrageenan Combination Results in Antiviral Synergy against SARS‐CoV‐1 and 2 in a Pseudoviral Model. Marine Drugs, 19(8), 418. 10.3390/md19080418 34436255 PMC8400000

[fft2119-bib-0006] Ambati, R. R. , Phang, S. M. , Ravi, S. , & Aswathanarayana, R. G. (2014). Astaxanthin: Sources, extraction, stability, biological activities and its commercial applications—A review. Marine Drugs, 12(1), 128–152. 10.3390/md12010128 24402174 PMC3917265

[fft2119-bib-0007] Andújar, I. , Ríos, J. L. , Giner, R. M. , & Recio, M. C. (2013). Pharmacological properties of shikonin—A review of literature since 2002. Planta Medica, 79(18), 1685–1697. 24155261 10.1055/s-0033-1350934

[fft2119-bib-0008] Aranow, C. (2011). Vitamin D and the immune system. Journal of Investigative Medicine, 59(6), 881–886. 21527855 10.231/JIM.0b013e31821b8755 PMC3166406

[fft2119-bib-0009] Arts, I. C. , van de Putte, B. , & Hollman, P. C. (2000). Catechin contents of foods commonly consumed in The Netherlands. 1. Fruits, vegetables, staple foods, and processed foods. Journal of Agricultural and Food Chemistry, 48(5), 1746–1751. 10820089 10.1021/jf000025h

[fft2119-bib-0010] Ashraf, W. , Latif, A. , Lianfu, Z. , Jian, Z. , Chenqiang, W. , Rehman, A. , Hussain, A. , Siddiquy, M. , & Karim, A. (2020). Technological advancement in the processing of lycopene: A review. Food Reviews International. 10.1080/87559129.2020.1749653

[fft2119-bib-0011] Astani, A. , Reichling, J. , & Schnitzler, P. (2010). Comparative study on the antiviral activity of selected monoterpenes derived from essential oils. Phytotherapy Research, 24(5), 673–679. 10.1002/ptr.2955 19653195 PMC7167768

[fft2119-bib-0012] Astani, A. , Reichling, J. , & Schnitzler, P. (2011). Screening for antiviral activities of isolated compounds from essential oils. Evidence‐Based Complementary and Alternative Medicine, 253643. 10.1093/ecam/nep187 20008902 PMC3096453

[fft2119-bib-0013] Atherton, J. G. , Kratzing, C. C. , & Fisher, A. (1978). The effect of ascorbic acid on infection of chick‐embryo ciliated tracheal organ cultures by coronavirus. Archives of Virology, 56(3), 195–199. 205194 10.1007/BF01317848 PMC7087159

[fft2119-bib-0014] Autier, P. , Mullie, P. , Macacu, A. , Dragomir, M. , Boniol, M. , Coppens, K. , Pizot, C. , & Boniol, M. (2017). Effect of vitamin D supplementation on non‐skeletal disorders: A systematic review of meta‐analyses and randomised trials. Lancet Diabetes & Endocrinology, 5(12), 986–1004. 10.1016/S2213-8587(17)30357-1 29102433

[fft2119-bib-0290] Bae, W.‐Y. , Kim, H.‐Y. , Choi, K.‐S. , Chang, K. H. , Hong, Y.‐H. , Eun, J. , Lee, N.‐K. , Paik, H.‐D. (2019). Investigation of Brassica juncea, Forsythia suspensa, and Inula britannica: phytochemical properties, antiviral effects, and safety. BMC Complementary and Alternative Medicine, 19(1), 10.1186/s12906-019-2670-x PMC6737602 31510997

[fft2119-bib-0016] Baharoon, S. , & Memish, Z. A. (2019). MERS‐CoV as an emerging respiratory illness: A review of prevention methods. Travel Medicine and Infectious Disease, 32, 101520. 10.1016/j.tmaid.2019.101520 PMC7110694 31730910

[fft2119-bib-0017] Barba, F. J. , Nikmaram, N. , Roohinejad, S. , Khelfa, A. , Zhu, Z. , & Koubaa, M. (2016). Bioavailability of glucosinolates and their breakdown products: Impact of processing. Frontiers in Nutrition, 3, 24. 10.3389/fnut.2016.00024 27579302 PMC4985713

[fft2119-bib-0018] Barton, C. , Kouokam, J. C. , Lasnik, A. B. , Foreman, O. , Cambon, A. , Brock, G. , Montefiori, D. C. , Vojdani, F. , Mccormick, A. A. , O'keefe, B. R. , & Palmer, K. E. (2014). Activity of and effect of subcutaneous treatment with the broad‐spectrum antiviral lectin Griffithsin in two laboratory rodent models. Antimicrobial Agents and Chemotherapy, 58(1), 120–127. 10.1128/Aac.01407-13 24145548 PMC3910741

[fft2119-bib-0019] Baum, M. K. , Miguez‐Burbano, M. J. , Campa, A. , & Shor‐Posner, G. (2000). Selenium and interleukins in persons infected with human immunodeficiency virus type 1. Journal of Infectious Diseases, 182, S69–S73. 10.1086/315911 10944486

[fft2119-bib-0020] Bekut, M. , Brkic, S. , Kladar, N. , Dragovic, G. , Gavaric, N. , & Bozin, B. (2018). Potential of selected Lamiaceae plants in anti(retro)viral therapy. Pharmacological Research, 133, 301–314. 10.1016/j.phrs.2017.12.016 29258916 PMC7129285

[fft2119-bib-0021] Belorkar, S. A. , & Gupta, A. K. (2016). Oligosaccharides: a boon from nature's desk. Amb Express, 6(1), 82. 27699701 10.1186/s13568-016-0253-5 PMC5047869

[fft2119-bib-0022] Bergman, P. , Lindh, A. U. , Bjorkhem‐Bergman, L. , & Lindh, J. D. (2013). Vitamin D and respiratory tract infections: A systematic review and meta‐analysis of randomized controlled trials. Plos One, 8(6), e65835. 10.1371/journal.pone.0065835 PMC3686844 23840373

[fft2119-bib-0023] Bhatia, L. , Sharma, A. , Bachheti, R. K. , & Chandel, A. K. (2019). Lignocellulose derived functional oligosaccharides: Production, properties, and health benefits. Preparative Biochemistry & Biotechnology, 49(8), 744–758. 10.1080/10826068.2019.1608446 31050587

[fft2119-bib-0024] Biancatelli, R. M. L. C. , Berrill, M. , Catravas, J. D. , & Marik, P. E. (2020). Quercetin and vitamin C: An experimental, synergistic therapy for the prevention and treatment of SARS‐CoV‐2 related disease (COVID‐19). Frontiers in Immunology, 11. 10.3389/fimmu.2020.01451 32636851 PMC7318306

[fft2119-bib-0025] Bleibtreu, A. , Bertine, M. , Bertin, C. , Houhou‐Fidouh, N. , & Visseaux, B. (2019). Focus on Middle East respiratory syndrome coronavirus (MERS‐CoV). Medecine et maladies infectieuses, 50(3), 243–251. 31727466 10.1016/j.medmal.2019.10.004 PMC7125975

[fft2119-bib-0026] Blot, M. , Bour, J.‐B. , Quenot, J. P. , Bourredjem, A. , Nguyen, M. , Guy, J. , Monier, S. , Georges, M. , Large, A. , Dargent, A. , Guilhem, A. , Mouries‐Martin, S. , Barben, J. , Bouhemad, B. , Charles, P.‐E. , Chavanet, P. , Binquet, C. , Piroth, L. , Andreu, P. , … Putot, A. (2020). The dysregulated innate immune response in severe COVID‐19 pneumonia that could drive poorer outcome. Journal of Translational Medicine, 18(1), 1–14. 33272291 10.1186/s12967-020-02646-9 PMC7711269

[fft2119-bib-0027] Boergeling, Y. , & Ludwig, S. (2017). Targeting a metabolic pathway to fight the flu. Febs Journal, 284(2), 218–221. 10.1111/febs.13997 28121076

[fft2119-bib-0028] Borges, A. , Abreu, A. C. , Ferreira, C. , Saavedra, M. J. , Simoes, L. C. , & Simoes, M. (2015). Antibacterial activity and mode of action of selected glucosinolate hydrolysis products against bacterial pathogens. Journal of Food Science and Technology, 52(8), 4737–4748. 10.1007/s13197-014-1533-1 26243895 PMC4519465

[fft2119-bib-0285] Bouazzi, S. , Jmii, H. , El Mokni, R. , Faidi, K. , Falconieri, D. , Piras, A. , Jaïdane, H. , Porcedda, S. , Hammami, S. (2018). Cytotoxic and antiviral activities of the essential oils from Tunisian Fern, Osmunda regalis. South African Journal of Botany, 118, 52–57. 10.1016/j.sajb.2018.06.015

[fft2119-bib-0029] Britton, G. (2020). Carotenoid research: History and new perspectives for chemistry in biological systems. Biochimica Et Biophysica Acta‐Molecular and Cell Biology of Lipids, 1865(11), 158699. 32205211 10.1016/j.bbalip.2020.158699

[fft2119-bib-0030] Burt, S. (2004). Essential oils: Their antibacterial properties and potential applications in foods—A review. International Journal of Food Microbiology, 94(3), 223–253. 15246235 10.1016/j.ijfoodmicro.2004.03.022

[fft2119-bib-0031] Calder, P. C. , Carr, A. C. , Gombart, A. F. , & Eggersdorfer, M. (2020). Optimal nutritional status for a well‐functioning immune system is an important factor to protect against viral infections. Nutrients, 12(4), 1181. 10.3390/nu12041181 PMC7230749 32340216

[fft2119-bib-0032] Campione, E. , Lanna, C. , Cosio, T. , Rosa, L. , Conte, M. P. , Iacovelli, F. , Romeo, A. , Falconi, M. , Del Vecchio, C. , Franchin, E. , Lia, M. S. , Minieri, M. , Chiaramonte, C. , Ciotti, M. , Nuccetelli, M. , Terrinoni, A. , Bernardini, S. , Coppeda, L. , Magrini, A. , … Bianchi, L. (2020). Lactoferrin as potential supplementary nutraceutical agent in COVID‐19 patients: in vitro and in vivo preliminary evidences. BioRxiv. 10.1101/2020.08.11.244996

[fft2119-bib-0033] Cegolon, L. , Mirandola, M. , Salaris, C. , Salvati, M. V. , Mastrangelo, G. , & Salata, C. (2021). Hypothiocyanite and hypothiocyanite/lactoferrin mixture exhibit virucidal activity in vitro against SARS‐CoV‐2. Pathogens, 10(2), 233. 33669635 10.3390/pathogens10020233 PMC7922920

[fft2119-bib-0034] Chainani‐Wu, N. (2003). Safety and anti‐inflammatory activity of curcumin: A component of tumeric (Curcuma longa). The Journal of Alternative & Complementary Medicine, 9(1), 161–168. 12676044 10.1089/107555303321223035

[fft2119-bib-0035] Chang, S. K. , Alasalvar, C. , & Shahidi, F. (2016). Review of dried fruits: Phytochemicals, antioxidant efficacies, and health benefits. Journal of Functional Foods, 21, 113–132.

[fft2119-bib-0037] Chen, X. Y. , Han, W. W. , Zhao, X. , Tang, W. , & Wang, F. H. (2019). Epirubicin‐loaded marine carrageenan oligosaccharide capped gold nanoparticle system for pH‐triggered anticancer drug release. Scientific Reports, 9, 6754. 31043709 10.1038/s41598-019-43106-9 PMC6494808

[fft2119-bib-0038] Chen, X. , Han, W. , Wang, G. , & Zhao, X. (2020). Application prospect of polysaccharides in the development of anti‐novel coronavirus drugs and vaccines. International Journal of Biological Macromolecules, 164, 331–343. 32679328 10.1016/j.ijbiomac.2020.07.106 PMC7358770

[fft2119-bib-0039] Cheng, R. Z. (2020). Can early and high intravenous dose of vitamin C prevent and treat coronavirus disease 2019 (COVID‐19)? Medicine in Drug Discovery, 5, 100028. 32328576 10.1016/j.medidd.2020.100028 PMC7167497

[fft2119-bib-0040] Choi, H.‐J. , Song, J.‐H. , Ahn, Y.‐J. , Baek, S.‐H. , & Kwon, D.‐H. (2009). Antiviral activities of cell‐free supernatants of yogurts metabolites against some RNA viruses. European Food Research and Technology, 228(6), 945–950.

[fft2119-bib-0041] Chow, K. Y. C. , Hon, C. C. , Hui, R. K. H. , Wong, R. T. Y. , Yip, C. W. , Zeng, F. , & Leung, F. C. C. (2003). Molecular advances in severe acute respiratory syndrome‐associated coronavirus (SARS‐CoV). Genomics, Proteomics & Bioinformatics, 1(4), 247–262. 10.1016/S1672-0229(03)01031-3 PMC5172416 15629054

[fft2119-bib-0042] Chowdhury, A. I. (2020). Role and effects of micronutrients supplementation in immune system and SARS‐Cov‐2 (COVID‐19). Asian Journal of Immunology, 47–55.

[fft2119-bib-0045] de Carvalho, C. A. M. , da Rocha Matos, A. , Caetano, B. C. , de Sousa Junior, I. P. , da Costa Campos, S. P. , Geraldino, B. R. , Barros, C. A. , de Almeida, M. A. P. , Rocha, V. P. , da Silva, A. M. V. , Melgaço, J. G. , da Costa Neves, P. C. , da Costa Barros, T. A. , Bom, A. P. D. A. , Siqueira, M. M. , Missailidis, S. , & Gonçalves, R. B. (2020). In vitro inhibition of SARS‐CoV‐2 infection by bovine lactoferrin. BioRxiv. 10.1101/2020.05.13.093781

[fft2119-bib-0046] De la Fuente, M. , Hernanz, A. , Guayerbas, N. , Victor, V. M. , & Arnalich, F. (2008). Vitamin E ingestion improves several immune functions in elderly men and women. Free Radical Research, 42(3), 272–280. 10.1080/10715760801898838 18344122

[fft2119-bib-0047] De Logu, A. , Loy, G. , Pellerano, M. L. , Bonsignore, L. , & Schivo, M. L. (2000). Inactivation of HSV‐1 and HSV‐2 and prevention of cell‐to‐cell virus spread by Santolina insularis is essential oil. Antiviral Research, 48(3), 177–185. 10.1016/S0166-3542(00)00127-3 11164504

[fft2119-bib-0043] da Mata, E. C. G. , Mourao, C. B. F. , Rangel, M. , & Schwartz, E. F. (2017). Antiviral activity of animal venom peptides and related compounds. Journal of Venomous Animals and Toxins Including Tropical Diseases, 23, 3. 10.1186/s40409-016-0089-0 PMC5217322 28074089

[fft2119-bib-0044] da Silva, P. G. , Nascimento, M. S. J. , Soares, R. R. G. , Sousa, S. I. V. , & Mesquita, J. R. (2020). Airborne spread of infectious SARS‐CoV‐2: Moving forward using lessons from SARS‐CoV and MERS‐CoV. Science of the Total Environment, 764, 142802. 10.1016/j.scitotenv.2020.142802 PMC7543729 33071145

[fft2119-bib-0278] del Valle Mendoza, J. , Pumarola, T. , Gonzales, L. A. , del Valle, L. J. (2014). Antiviral activity of maca (Lepidium meyenii) against human influenza virus. Asian Pacific Journal of Tropical Medicine, 7, S415–S420. 10.1016/s1995-7645(14)60268-6 25312160

[fft2119-bib-0048] Decker, J. S. , Menacho‐Melgar, R. , & Lynch, M. D. (2020). Low‐cost, large‐scale production of the anti‐viral lectin Griffithsin. Frontiers in Bioengineering and Biotechnology, 8, 1020. 32974328 10.3389/fbioe.2020.01020 PMC7471252

[fft2119-bib-0049] Degnah, A. A. , Al‐Amri, S. S. , Hassan, A. M. , Almasoud, A. S. , Mousa, M. , Almahboub, S. A. , Alhabbab, R. Y. , Mirza, A. A. , Hindawi, S. I. , Alharbi, N. K. , Azhar, E. I. , & Hashem, A. M. (2020). Seroprevalence of MERS‐CoV in healthy adults in western Saudi Arabia, 2011–2016. Journal of Infection and Public Health, 13(5), 697–703. 10.1016/j.jiph.2020.01.001 32005618 PMC7104088

[fft2119-bib-0050] Desbarats, J. (2020). Pyridoxal 5′‐phosphate to mitigate immune dysregulation and coagulopathy in COVID‐19. 10.20944/preprints202005.0144.v1

[fft2119-bib-0051] Dias, C. , Aires, A. , Bennett, R. N. , Rosa, E. A. , & Saavedra, M. J. (2012). First study on antimicriobial activity and synergy between isothiocyanates and antibiotics against selected Gram‐negative and Gram‐positive pathogenic bacteria from clinical and animal source. Medicinal Chemistry, 8(3), 474–480. 10.2174/1573406411208030474 22530889

[fft2119-bib-0052] Dobrange, E. , Peshev, D. , Loedolff, B. , & Van den Ende, W. (2019). Fructans as immunomodulatory and antiviral agents: The case of Echinacea. Biomolecules, 9(10), 615. 10.3390/biom9100615 PMC6843407 31623122

[fft2119-bib-0054] dos Santos, L. M. J. (2020). Can vitamin B12 be an adjuvant to COVID‐19 treatment? GSC Biological and Pharmaceutical Sciences, 11(3), 001–005.

[fft2119-bib-0055] Doty, R. L. (2019). Treatments for smell and taste disorders: A critical review. Handbook of clinical neurology (Vol. 164, pp. 455–479). Elsevier. 31604562 10.1016/B978-0-444-63855-7.00025-3

[fft2119-bib-0056] Duda‐Chodak, A. , Lukasiewicz, M. , Zięć, G. , Florkiewicz, A. , & Filipiak‐Florkiewicz, A. (2020). Covid‐19 pandemic and food: Present knowledge, risks, consumers fears and safety. Trends in Food Science & Technology, 105, 145–160. 32921922 10.1016/j.tifs.2020.08.020 PMC7480472

[fft2119-bib-0057] Duijker, G. , Bertsias, A. , Symvoulakis, E. K. , Moschandreas, J. , Malliaraki, N. , Derdas, S. P. , Tsikalas, G. K. , Katerinopoulos, H. E. , Pirintsos, S. A. , Sourvinos, G. , Castanas, E. , & Lionis, C. (2015). Reporting effectiveness of an extract of three traditional Cretan herbs on upper respiratory tract infection: Results from a double‐blind randomized controlled trial. Journal of Ethnopharmacology, 163, 157–166. 25645191 10.1016/j.jep.2015.01.030 PMC7127758

[fft2119-bib-0287] Duschatzky, C. B. , Possetto, M. L. , Talarico, L. B. , García, C. C. , Michis, F. , Almeida, N. V. , de Lampasona, M. P. , Schuff, C. , Damonte, E. B. (2005). Evaluation of Chemical and Antiviral Properties of Essential Oils from South American Plants. Antiviral Chemistry and Chemotherapy, 16(4), 247–251. 10.1177/095632020501600404 16130522

[fft2119-bib-0058] El Hadi , M. A. M. , Zhang, F. J. , Wu, F. F. , Zhou, C. H. , & Tao, J. (2013). Advances in fruit aroma volatile research. Molecules, 18(7), 8200–8229. 10.3390/molecules18078200 23852166 PMC6270112

[fft2119-bib-0060] El‐Sekaily, A. , Helal, M. , & Saad, A. (2020). Enhancement of immune tolerance of COVID‐19 patients might be achieved with alginate supplemented therapy. International Journal of Cancer and Biomedical Research, 4, 21‐26.

[fft2119-bib-0061] Farshi, P. , Kaya, E. C. , Hashempour‐Baltork, F. , & Khosravi‐Darani, K. (2020). A comprehensive review on the effect of plant metabolites on coronaviruses: focusing on their molecular docking score and IC_50_ values. *Preprints*, 2020050295. doi: 10.20944/preprints202005.0295.v1 10.2174/1389557521666210831152511 34488609

[fft2119-bib-0269] Fowler III Alpha A , Kim, C. , Lepler, L. , Malhotra, R. , Debesa, O. , Natarajan, R. , Fisher, B. J. , Syed, A. , DeWilde, C. , Priday, A. , Kasirajan, V. (2017). Intravenous vitamin C as adjunctive therapy for enterovirus/rhinovirus induced acute respiratory distress syndrome. World Journal of Critical Care Medicine, 6(1), 85. 10.5492/wjccm.v6.i1.85 28224112 PMC5295174

[fft2119-bib-0062] Fraga, C. G. , Croft, K. D. , Kennedy, D. O. , & Tomás‐Barberán, F. A. (2019). The effects of polyphenols and other bioactives on human health. Food & Function, 10(2), 514–528. 30746536 10.1039/c8fo01997e

[fft2119-bib-0063] Gabryszewski, S. J. , Bachar, O. , Dyer, K. D. , Percopo, C. M. , Killoran, K. E. , Domachowske, J. B. , & Rosenberg, H. F. (2011). Lactobacillus‐mediated priming of the respiratory mucosa protects against lethal pneumovirus infection. The Journal of Immunology, 186(2), 1151–1161. 21169550 10.4049/jimmunol.1001751 PMC3404433

[fft2119-bib-0064] Galmes, S. , Serra, F. , & Palou, A. (2018). Vitamin E metabolic effects and genetic variants: A challenge for precision nutrition in obesity and associated disturbances. Nutrients, 10(12), 1919. 10.3390/nu10121919 PMC6316334 30518135

[fft2119-bib-0065] Garbati, M. A. , Fagbo, S. F. , Fang, V. J. , Skakni, L. , Joseph, M. , Wani, T. A. , Cowling, B. J. , Peiris, M. , & Hakawi, A. (2016). A comparative study of clinical presentation and risk factors for adverse outcome in patients hospitalised with acute respiratory disease due to MERS coronavirus or other causes. Plos One, 11(11), e0165978. 27812197 10.1371/journal.pone.0165978 PMC5094725

[fft2119-bib-0066] García‐Salido, A. , Antón, J. , Martínez‐Pajares, J. D. , Garcia, G. G. , Cortés, B. G. , Tagarro, A. , & Grupo de trabajo de la Asociación Española de Pediatría para el Síndrome Inflamatorio Multisistémico Pediátrico vinculado a SARS‐CoV (2020a). Documento español de consenso sobre diagnóstico, estabilización y tratamiento del síndrome inflamatorio multisistémico pediátrico vinculado a SARS‐CoV‐2 (SIM‐PedS). *Anales de pediatria*, *94*(2), 116.e1–116.e11. 10.1016/j.anpedi.2020.09.005 PMC7604157 33132066

[fft2119-bib-0067] García‐Salido, A. , Antón, J. , Martínez‐Pajares, J. D. , Giralt Garcia, G. , Gómez Cortés, B. , & Tagarro, A. (2020b). Documento español de consenso sobre diagnóstico, estabilización y tratamiento del síndrome inflamatorio multisistémico pediátrico vinculado a SARS‐CoV‐2 (SIM‐PedS). Anales de Pediatría. 10.1016/j.anpedi.2020.09.005 PMC7604157 33132066

[fft2119-bib-0068] Gasmi, A. , Noor, S. , Tippairote, T. , Dadar, M. , Menzel, A. , & Bjorklund, G. (2020). Individual risk management strategy and potential therapeutic options for the COVID‐19 pandemic. Clinical Immunology, 215, 108409 32276137 10.1016/j.clim.2020.108409 PMC7139252

[fft2119-bib-0289] Gavanji, S. , Sayedipour, S. S. , Larki, B. , Bakhtari, A. (2015). Antiviral activity of some plant oils against herpes simplex virus type 1 in Vero cell culture. Journal of Acute Medicine, 5(3), 62–68. 10.1016/j.jacme.2015.07.001

[fft2119-bib-0069] Gharibzahedi, S. M. T. , & Jafari, S. M. (2017). The importance of minerals in human nutrition: Bioavailability, food fortification, processing effects and nanoencapsulation. Trends in Food Science & Technology, 62, 119–132. 10.1016/j.tifs.2017.02.017

[fft2119-bib-0070] Ghendon, Y. , Markushin, S. , Vasiliev, Y. , Akopova, I. , Koptiaeva, I. , Krivtsov, G. , Borisova, O. , Ahmatova, N. , Kurbatova, E. , Mazurina, S. , & Gervazieva, V. (2009). Evaluation of properties of chitosan as an adjuvant for inactivated influenza vaccines administered parenterally. Journal of Medical Virology, 81(3), 494–506. 10.1002/jmv.21415 19152418

[fft2119-bib-0071] Giannis, D. , Ziogas, I. A. , & Gianni, P. (2020). Coagulation disorders in coronavirus infected patients: COVID‐19, SARS‐CoV‐1, MERS‐CoV and lessons from the past. Journal of Clinical Virology, 104362. 32305883 10.1016/j.jcv.2020.104362 PMC7195278

[fft2119-bib-0072] Gilling, D. H. , Kitajima, M. , Torrey, J. R. , & Bright, K. R. (2014). Antiviral efficacy and mechanisms of action of oregano essential oil and its primary component carvacrol against murine norovirus. Journal of Applied Microbiology, 116(5), 1149–1163. 24779581 10.1111/jam.12453

[fft2119-bib-0074] Giuffrida, D. , Zoccali, M. , & Mondello, L. (2020). Recent developments in the carotenoid and carotenoid derivatives chromatography‐mass spectrometry analysis in food matrices. Trends in Analytical Chemistry, 132, 116047.

[fft2119-bib-0075] Gonzalez, S. (2020). Dietary bioactive compounds and human health and disease. Nutrients, 12(2), 348. 10.3390/nu12020348 PMC7071229 32013067

[fft2119-bib-0076] González‐Chávez, S. A. , Arévalo‐Gallegos, S. , & Rascón‐Cruz, Q. (2009). Lactoferrin: Structure, function and applications. International Journal of Antimicrobial Agents, 33(4), 301–e1. 18842395 10.1016/j.ijantimicag.2008.07.020

[fft2119-bib-0077] Gordon, M. , Guralnik, M. , Kaneko, Y. , Mimura, T. , Goodgame, J. , DeMarzo, C. , Pierce, D. , Baker, M. , & Lang, W. (1995). A phase II controlled study of a combination of the immune modulator, lentinan, with didanosine (DDI) in HIV patients with CD4 cells of 200‐500/MM(3). Journal of Medicine, 26(5–6), 193–207. 8721897

[fft2119-bib-0078] Goudarzi, M. , Garavand, F. , Madadlou, A. , & Fogliano, V. (2020). Food protein‐derived antihypertensive peptides in the COVID‐19 pandemic: Friends of foes? Journal of Hypertension. 10.1097/HJH.0000000000002534 PMC7282401 32472779

[fft2119-bib-0079] Goudarzi, M. , & Madadlou, A. (2013). Influence of whey protein and its hydrolysate on prehypertension and postprandial hyperglycaemia in adult men. International Dairy Journal, 33(1), 62–66. 10.1016/j.idairyj.2013.06.006

[fft2119-bib-0080] Goudarzi, M. , Madadlou, A. , Mousavi, M. E. , & Emam‐Djomeh, Z. (2012). Optimized preparation of ACE‐inhibitory and antioxidative whey protein hydrolysate using response surface method. Dairy Science & Technology, 92(6), 641–653. 10.1007/s13594-012-0081-6

[fft2119-bib-0081] Grant, W. B. , Lahore, H. , McDonnell, S. L. , Baggerly, C. A. , French, C. B. , Aliano, J. L. , & Bhattoa, H. P. (2020). Evidence that vitamin D supplementation could reduce risk of influenza and COVID‐19 infections and deaths. Nutrients, 12(4), 988. 10.3390/nu12040988 PMC7231123 32252338

[fft2119-bib-0082] Guo, Q. , Sun, X. , Zhang, Z. , Zhang, L. , Yao, G. , Li, F. , Yang, X. , Song, L. , & Jiang, G. (2014). The effect of Astragalus polysaccharide on the Epstein‐Barr virus lytic cycle. Acta Virologica, 58(1), 76–80. 10.4149/av_2014_01_76 24717032

[fft2119-bib-0083] Gupta, P. K. , Agrawal, P. , Hegde, P. , Shankarnarayan, N. , Vidyashree, S. , Singh, S. A. , & Ahuja, S. (2016). Xylooligosaccharide—A valuable material from waste to taste: A review. Journal of Environmental Research and Development, 10(3), 555.

[fft2119-bib-0084] Gutierrez‐Castrellon, P. , Lopez‐Velazquez, G. , Diaz‐Garcia, L. , Jimenez‐Gutierrez, C. , Mancilla‐Ramirez, J. , Estevez‐Jimenez, J. , & Parra, M. (2014). Diarrhea in preschool children and Lactobacillus reuteri: A randomized controlled trial. Pediatrics, 133(4), e904–e909. 24639271 10.1542/peds.2013-0652

[fft2119-bib-0085] Hamid, S. , Mir, M. Y. , & Rohela, G. K. (2020). Novel coronavirus disease (COVID‐19): A pandemic (epidemiology, pathogenesis and potential therapeutics). New Microbes and New Infections, 35, 100679. 32322401 10.1016/j.nmni.2020.100679 PMC7171518

[fft2119-bib-0086] Hegazy, G. E. , Abu‐Serie, M. M. , Abo‐Elela, G. M. , Ghozlan, H. , Sabry, S. A. , Soliman, N. A. , & Abdel‐Fattah, Y. R. (2020). In vitro dual (anticancer and antiviral) activity of the carotenoids produced by haloalkaliphilic archaeon Natrialba sp. M6. Scientific Reports, 10(1), 5986. 32249805 10.1038/s41598-020-62663-y PMC7136267

[fft2119-bib-0087] Hellinger, R. , & Gruber, C. W. (2019). Peptide‐based protease inhibitors from plants. Drug Discovery Today, 24(9), 1877–1889. 31170506 10.1016/j.drudis.2019.05.026 PMC6753016

[fft2119-bib-0088] Hemila, H. (1997). Vitamin C intake and susceptibility to pneumonia. Pediatric Infectious Disease Journal, 16(9), 836–837. 10.1097/00006454-199709000-00003 9306475

[fft2119-bib-0089] Hemila, H. (2017). Vitamin C and infections. Nutrients, 9(4), 339. 10.3390/nu9040339 PMC5409678 28353648

[fft2119-bib-0090] Hemila, H. , Petrus, E. J. , Fitzgerald, J. T. , & Prasad, A. (2016). Zinc acetate lozenges for treating the common cold: An individual patient data meta‐analysis. British Journal of Clinical Pharmacology, 82(5), 1393–1398. 10.1111/bcp.13057 27378206 PMC5061795

[fft2119-bib-0091] Hester, S. N. , Chen, X. , Li, M. , Monaco, M. H. , Comstock, S. S. , Kuhlenschmidt, T. B. , Kuhlenschmidt, M. S. , & Donovan, S. M. (2013). Human milk oligosaccharides inhibit rotavirus infectivity in vitro and in acutely infected piglets. British Journal of Nutrition, 110(7), 1233–1242. 10.1017/S0007114513000391 23442265

[fft2119-bib-0286] Ho, T. T. , Tran, Q. T. N. , Chai, C. L. L. (2018). The polypharmacology of natural products. Future Medicinal Chemistry, 10(11), 1361–1368. 10.4155/fmc-2017-0294 29673257

[fft2119-bib-0092] Horbowicz, M. (2003). The occurrence, role and contents of glucosinolates in Brassica vegetables. Vegetable Crops Research Bulletin, 58, 23–40.

[fft2119-bib-0093] Hu, N. , Li, Q.‐B. , & Zou, S. Y. (2018). Effect of vitamin A as an adjuvant therapy for pneumonia in children: A meta‐analysis. *Zhongguo Dang Dai Er Ke Za Zhi*, 20(2),146–153. 10.7499/j.issn.1008-8830.2018.02.013 PMC7389232 29429465

[fft2119-bib-0094] Hu, Y. , Meng, X. , Zhang, F. , Xiang, Y. , & Wang, J. (2021). The in vitro antiviral activity of lactoferrin against common human coronaviruses and SARS‐CoV‐2 is mediated by targeting the heparan sulfate co‐receptor. Emerging Microbes & Infections, 10(1), 317–330. 33560940 10.1080/22221751.2021.1888660 PMC7919907

[fft2119-bib-0095] Huang, C. , Wang, Y. , Li, X. , Ren, L. , Zhao, J. , Hu, Y. , Zhang, L. , Fan, G. , Xu, J. , Gu, X. , Cheng, Z. , Yu, T. , Xia, J. , Wei, Y. , Wu, W. , Xie, X. , Yin, W. , Li, H. , Liu, M. , … Cao, B. (2020). Clinical features of patients infected with 2019 novel coronavirus in Wuhan, China. Lancet, 395(10223), 497–506. 10.1016/S0140-6736(20)30183-5 31986264 PMC7159299

[fft2119-bib-0096] Huang, G. L. , Chen, X. , & Huang, H. L. (2016). Chemical modifications and biological activities of polysaccharides. Current Drug Targets, 17(15), 1799–1803. 10.2174/1389450117666160502151004 27138762

[fft2119-bib-0097] Iakovidis, I. , Delimaris, I. , & Piperakis, S. M. (2011). Copper and its complexes in medicine: A biochemical approach. Molecular Biology International, 2011;2011:594529. 22091409 10.4061/2011/594529 PMC3195324

[fft2119-bib-0098] Islam, M. T. , da Mata, A. M. O. F. , de Aguiar, R. P. S. , Paz, M. F. C. J. , de Alencar, M. V. O. B. , Ferreira, P. M. P. , & Melo‐Cavalcante, A. A. D. (2016). Therapeutic potential of essential oils focusing on diterpenes. Phytotherapy Research, 30(9), 1420–1444. 10.1002/ptr.5652 27307034

[fft2119-bib-0099] Jaber, N. , Al‐Remawi, M. , Al‐Akayleh, F. , Al‐Muhtaseb, N. , Al‐Adham, I. S. , & Collier, P. J. (2021). A review of the antiviral activity of Chitosan, including patented applications and its potential use against COVID‐19. Journal of Applied Microbiology. 10.1111/jam.15202 PMC8447037 34218488

[fft2119-bib-0100] Jackwood, M. W. , Rosenbloom, R. , Petteruti, M. , Hilt, D. A. , McCall, A. W. , & Williams, S. M. (2010). Avian coronavirus infectious bronchitis virus susceptibility to botanical oleoresins and essential oils in vitro and in vivo. Virus Research, 149(1), 86–94. 10.1016/j.virusres.2010.01.006 20096315 PMC7114412

[fft2119-bib-0101] Jee, J. , Hoet, A. E. , Azevedo, M. P. , Vlasova, A. N. , Loerch, S. C. , Pickworth, C. L. , Hanson, J. , & Saif, L. J. (2013). Effects of dietary vitamin A content on antibody responses of feedlot calves inoculated intramuscularly with an inactivated bovine coronavirus vaccine. American Journal of Veterinary Research, 74(10), 1353–1362. 24066921 10.2460/ajvr.74.10.1353

[fft2119-bib-0102] Jeffery, L. E. , Burke, F. , Mura, M. , Zheng, Y. , Qureshi, O. S. , Hewison, M. , Walker, L. S. K. , Lammas, D. A. , Raza, K. , & Sansom, D. M. (2009). 1, 25‐Dihydroxyvitamin D3 and IL‐2 combine to inhibit T cell production of inflammatory cytokines and promote development of regulatory T cells expressing CTLA‐4 and FoxP3. The Journal of Immunology, 183(9), 5458–5467. 19843932 10.4049/jimmunol.0803217 PMC2810518

[fft2119-bib-0103] Ji, J. , Wang, L. C. , Wu, H. , & Luan, H.‐M. (2011). Bio‐function summary of marine oligosaccharides. International Journal of Biology, 3(1), 74–86.

[fft2119-bib-0104] Jo, S. , Kim, S. , Shin, D. H. , Kim, M.‐S. (2020). Inhibition of SARS‐CoV 3CL protease by flavonoids. Journal of Enzyme Inhibition and Medicinal Chemistry, 35(1), 145–151. 10.1080/14756366.2019.1690480 31724441 PMC6882434

[fft2119-bib-0105] Johnson, I. T. (2002). Glucosinolates: bioavailability and importance to health. International Journal for Vitamin and Nutrition Research, 72(1), 26–31. 11887749 10.1024/0300-9831.72.1.26

[fft2119-bib-0106] Jones, H. D. , Yoo, J. , Crother, T. R. , Kyme, P. , Ben‐Shlomo, A. , Khalafi, R. , Tseng, C. W. , Parks, W. C. , Arditi, M. , Liu, G. Y. , & Shimada, K. (2015). Nicotinamide exacerbates hypoxemia in ventilator‐induced lung injury independent of neutrophil infiltration. Plos One, 10(4), e0123460. 25875775 10.1371/journal.pone.0123460 PMC4395431

[fft2119-bib-0107] Kandeil, A. , Gomaa, M. , Shehata, M. , El‐Taweel, A. , Kayed, A. E. , Abiadh, A. , Jrijer, J. , Moatasim, Y. , Kutkat, O. , Bagato, O. , Mahmoud, S. , Mostafa, A. , El‐Shesheny, R. , Perera, R. A. , Ko, R. L. , Hassan, N. , Elsokary, B. , Allal, L. , Saad, A. , … Kayali, G. (2019). Middle East respiratory syndrome coronavirus infection in non‐camelid domestic mammals. Emerging Microbes & Infections, 8(1), 103–108. 30866764 10.1080/22221751.2018.1560235 PMC6455111

[fft2119-bib-0108] Keil, S. D. , Bowen, R. , & Marschner, S. (2016). Inactivation of Middle East respiratory syndrome coronavirus (MERS‐C o V) in plasma products using a riboflavin‐based and ultraviolet light‐based photochemical treatment. Transfusion, 56(12), 2948–2952. 27805261 10.1111/trf.13860 PMC7169765

[fft2119-bib-0109] Keyhan, S. O. , Fallahi, H. R. , & Cheshmi, B. (2020). Dysosmia and dysgeusia due to the 2019 novel coronavirus: A hypothesis that needs further investigation. Maxillofacial Plastic and Reconstructive Surgery, 42(1), 9. 32289035 10.1186/s40902-020-00254-7 PMC7103905

[fft2119-bib-0272] Kim, T. K. , Lim, H. R. , Byun, J. S. (2020). Vitamin C supplementation reduces the odds of developing a common cold in Republic of Korea Army recruits: randomised controlled trial. BMJ Military Health, bmjmilitary–2019. 10.1136/bmjmilitary-2019-001384 32139409

[fft2119-bib-0110] Khan, A. A. , Gani, A. , Khanday, F. A. , & Masoodi, F. A. (2018). Biological and pharmaceutical activities of mushroom β‐glucan discussed as a potential functional food ingredient. Bioactive Carbohydrates and Dietary Fibre, 16, 1–13.

[fft2119-bib-0111] Khan, S. , El Morabet, R. , Khan, R. A. , Bindajam, A. , Alqadhi, S. , Alsubih, M. , & Khan, N. A. (2020). Where we missed? Middle East respiratory syndrome (MERS‐CoV) epidemiology in Saudi Arabia; 2012–2019. Science of The Total Environment, 747, 141369. 10.1016/j.scitotenv.2020.141369 PMC7398055 32791417

[fft2119-bib-0112] Koch, C. , Reichling, J. , Schneele, J. , & Schnitzler, P. (2008). Inhibitory effect of essential oils against herpes simplex virus type 2. Phytomedicine, 15(1–2), 71–78. 17976968 10.1016/j.phymed.2007.09.003

[fft2119-bib-0113] Koenighofer, M. , Lion, T. , Bodenteich, A. , Prieschl‐Grassauer, E. , Grassauer, A. , Unger, H. , Mueller, C. A. , & Fazekas, T. (2014). Carrageenan nasal spray in virus confirmed common cold: Individual patient data analysis of two randomized controlled trials. Multidisciplinary Respiratory Medicine, 9, 57. 25411637 10.1186/2049-6958-9-57 PMC4236476

[fft2119-bib-0116] Labro, M. T. (2012). Immunomodulatory effects of antimicrobial agents. Part I: Antibacterial and antiviral agents. Expert Review of Anti‐Infective Therapy, 10(3), 319–340. 10.1586/Eri.12.11 22397566

[fft2119-bib-0117] Lang, J. S. , Yang, N. , Deng, J. J. , Liu, K. T. , Yang, P. , Zhang, G. G. , & Jiang, C. Y. (2011). Inhibition of SARS pseudovirus cell entry by lactoferrin binding to heparan sulfate proteoglycans. Plos One, 6(8), e23710. 21887302 10.1371/journal.pone.0023710 PMC3161750

[fft2119-bib-0118] Laucirica, D. R. , Triantis, V. , Schoemaker, R. , Estes, M. K. , & Ramani, S. (2017). Milk oligosaccharides inhibit human rotavirus infectivity in MA104 cells. Journal of Nutrition, 147(9), 1709–1714. 10.3945/jn.116.246090 PMC5572490 28637685

[fft2119-bib-0119] Lechien, J. R. , Chiesa‐Estomba, C. M. , De Siati, D. R. , Horoi, M. , Le Bon, S. D. , Rodriguez, A. , Dequanter, D. , Blecic, S. , El Afia, F. , Distinguin, L. , Chekkoury‐Idrissi, Y. , Hans, S. , Delgado, I. L. , Calvo‐Henriquez, C. , Lavigne, P. , Falanga, C. , Barillari, M. R. , Cammaroto, G. , Khalife, M. , … Saussez, S. (2020). Olfactory and gustatory dysfunctions as a clinical presentation of mild‐to‐moderate forms of the coronavirus disease (COVID‐19): A multicenter European study. European Archives of Oto‐Rhino‐Laryngology, 277(8), 2251–2261. 10.1007/s00405-020-05965-1 32253535 PMC7134551

[fft2119-bib-0283] Lee, N.‐K. , Lee, J.‐H. , Lim, S.‐M. , Lee, K.A. , Kim, Y.B. , Chang, P.‐S. , Paik, H.‐D. (2014). Short communication: Antiviral activity of subcritical water extract of Brassica juncea against influenza virus A/H1N1 in nonfat milk. Journal of Dairy Science, 97(9), 5383–5386. 10.3168/jds.2014-8016 25022686

[fft2119-bib-0120] Lee, C. (2019). Griffithsin, a highly potent broad‐spectrum antiviral lectin from red algae: From discovery to clinical application. Marine Drugs, 17(10), 567. 10.3390/md17100567 PMC6835697 31590428

[fft2119-bib-0121] Lee, J. B. , Miyake, S. , Umetsu, R. , Hayashi, K. , Chijimatsu, T. , & Hayashi, T. (2012). Anti‐influenza A virus effects of fructan from Welsh onion (Allium fistulosum L.). Food Chemistry, 134(4), 2164–2168. 10.1016/j.foodchem.2012.04.016 23442670 PMC7173106

[fft2119-bib-0122] Lehtoranta, L. , Pitkäranta, A. , & Korpela, R. (2014). Probiotics in respiratory virus infections. European Journal of Clinical Microbiology & Infectious Diseases, 33(8), 1289–1302. 24638909 10.1007/s10096-014-2086-y PMC7088122

[fft2119-bib-0123] Lei, W.‐T. , Shih, P.‐C. , Liu, S.‐J. , Lin, C.‐Y. , & Yeh, T.‐L. (2017). Effect of probiotics and prebiotics on immune response to influenza vaccination in adults: A systematic review and meta‐analysis of randomized controlled trials. Nutrients, 9(11), 1175. 10.3390/nu9111175 PMC5707647 29077061

[fft2119-bib-0125] Li, S. , Chen, C. , Zhang, H. , Guo, H. , Wang, H. , Wang, L. , Zhang, X. , Hua, S. , Yu, J. , & Xiao, P. (2005). Identification of natural compounds with antiviral activities against SARS‐associated coronavirus. Antiviral Research, 67(1), 18–23. 10.1016/j.antiviral.2005.02.007 15885816 PMC7114104

[fft2119-bib-0124] Li, J. , Zhou, X. , Zhang, Y. , Zhong, F. , Lin, C. , McCormick, P. J. , Jiang, F. , Luo, J. , Zhou, H. , Wang, Q. , Fu, Y. , Duan, J. , & Zhang, J. (2021). Crystal structure of SARS‐CoV‐2 main protease in complex with the natural product inhibitor shikonin illuminates a unique binding mode. Science Bulletin. 10.1016/j.scib.2020.10.018 PMC7598899 33163253

[fft2119-bib-0266] Li, Z. , Liu, Y. , Fang, Z. , Yang, L. , Zhuang, M. , Zhang, Y. , Lv, H. (2019). Natural Sulforaphane From Broccoli Seeds Against Influenza A Virus Replication in MDCK Cells. Natural Product Communications, 14(6), 1934578X1985822. 10.1177/1934578x19858221

[fft2119-bib-0126] Lin, Y. S. , Lin, C. F. , Fang, Y. T. , Kuo, Y. M. , Liao, P. C. , Yeh, T. M. , Hwa, K. Y. , Shieh, C. C. K. , Yen, J. H. , Wang, H. J. , Su, I. J. , & Lei, H. Y. (2005). Antibody to severe acute respiratory syndrome (SARS)‐associated coronavirus spike protein domain 2 cross‐reacts with lung epithelial cells and causes cytotoxicity. Clinical & Experimental Immunology, 141(3), 500–508. 16045740 10.1111/j.1365-2249.2005.02864.x PMC1809466

[fft2119-bib-0128] Liu, A.‐L. , Liu, B. , Qin, H.‐L. , Lee, S. M. Y. , Wang, Y.‐T. , & Du, G.‐H. (2008). Anti‐influenza virus activities of flavonoids from the medicinal plant Elsholtzia rugulosa. Planta Medica, 74(08), 847–851. 18553272 10.1055/s-2008-1074558

[fft2119-bib-0129] Liu, B. , Li, M. , Zhou, Z. , Guan, X. , Xiang, Y. (2020). Can we use interleukin‐6 (IL‐6) blockade for coronavirus disease 2019 (COVID‐19)‐induced cytokine release syndrome (CRS)?. Journal of Autoimmunity, 111, 102452. 10.1016/j.jaut.2020.102452 32291137 PMC7151347

[fft2119-bib-0273] Loeb, M. , Dang, A. D. , Thiem, V. D. , Thanabalan, V. , Wang, B. , Nguyen, N. B. , Tran, H. T. M. , Luong, T. M. , Singh, P. , Smieja, M. , Maguire, J. , Pullenayegum, E. (2019). Effect of Vitamin D supplementation to reduce respiratory infections in children and adolescents in Vietnam: A randomized controlled trial. Influenza and Other Respiratory Viruses, 13(2), 176–183. 10.1111/irv.12615 30328294 PMC6379634

[fft2119-bib-0131] Loizzo, M. R. , Saab, A. M. , Tundis, R. , Statti, G. A. , Menichini, F. , Lampronti, I. , Gambari, R. , Cinatl, J. , & Doerr, H. W. (2008). Phytochemical analysis and in vitro antiviral activities of the essential oils of seven Lebanon species. Chemistry & Biodiversity, 5(3), 461–470. 10.1002/cbdv.200890045 18357554 PMC7161995

[fft2119-bib-0288] Luo, Z. , Liu, L.‐F. , Wang, X.‐H. , Li, W. , Jie, C. , Chen, H. , Wei, F.‐Q. , Lu, D.‐H. , Yan, C.‐Y. , Liu, B. , Kurihara, H. , Li, Y.‐F. , He, R.‐R. (2019). Epigoitrin, an Alkaloid From Isatis indigotica, Reduces H1N1 Infection in Stress‐Induced Susceptible Model in vivo and in vitro. Frontiers in Pharmacology, 10, 10.3389/fphar.2019.00078 PMC6374342 30792656

[fft2119-bib-0132] Madadlou, A. (2020). Food proteins are a potential resource for mining cathepsin L inhibitory drugs to combat SARS‐CoV‐2. European Journal of Pharmacology, 885, 173499. 32841639 10.1016/j.ejphar.2020.173499 PMC7443098

[fft2119-bib-0133] Manach, C. , Scalbert, A. , Morand, C. , Rémésy, C. , & Jiménez, L. (2004). Polyphenols: Food sources and bioavailability. The American Journal of Clinical Nutrition, 79(5), 727–747. 15113710 10.1093/ajcn/79.5.727

[fft2119-bib-0135] Martineau, A. R. , Jolliffe, D. A. , Greenberg, L. , Aloia, J. F. , Bergman, P. , Dubnov‐Raz, G. , Esposito, S. , Ganmaa, D. , Ginde, A. A. , Goodall, E. C. , Grant, C. C. , Janssens, W. , Jensen, M. E. , Kerley, C. P. , Laaksi, I. , Manaseki‐Holland, S. , Mauger, D. , Murdoch, D. R. , Neale, R. , … Hooper, R. L. (2019). Vitamin D supplementation to prevent acute respiratory infections: Individual participant data meta‐analysis. Health Technology Assessment, 23(2), 1–44. 10.3310/hta23020 PMC6369419 30675873

[fft2119-bib-0136] Martineau, A. , Jolliffe, D. A. , Hooper, R. , Greenberg, L. , Aloia, J. , Bergman, P. , Dubnov‐Raz, G. , Esposito, S. , Ganmaa, D. , Ginde, A. , Goodall, E. , Grant, C. , Griffiths, C. , Janssens, W. , Laaksi, I. , Manaseki‐Holland, S. , Mauger, D. , Murdoch, D. , Neale, R. , … Camargo, C. (2017). Vitamin D supplementation to prevent acute respiratory tract infections: Systematic review and meta‐analysis of individual participant data. Bmj‐British Medical Journal, 356, i6583. 10.1136/bmj.i6583 PMC5310969 28202713

[fft2119-bib-0137] McCutcheon, A. R. , Roberts, T. E. , Gibbons, E. , Ellis, S. M. , Babiuk, L. A. , Hancock, R. E. W. , & Towers, G. H. N. (1995). Antiviral screening of British Columbian medicinal plants. Journal of Ethnopharmacology, 49(2), 101–110. 8847882 10.1016/0378-8741(95)90037-3 PMC7131204

[fft2119-bib-0138] Menachery, V. D. , Mitchell, H. D. , Cockrell, A. S. , Gralinski, L. E. , Yount, B. L. , Graham, R. L. , Mcanarney, E. T. , Douglas, M. G. , Scobey, T. , Beall, A. , Dinnon, K. , Kocher, J. F. , Hale, A. E. , Stratton, K. G. , Waters, K. M. , & Baric, R. S. (2017). MERS‐CoV accessory ORFs play key role for infection and pathogenesis. MBio, 8(4), e00665‐17. 10.1128/mBio.00665-17 PMC5565963 28830941

[fft2119-bib-0139] Meydani, S. N. , Leka, L. S. , Fine, B. C. , Dallal, G. E. , Keusch, G. T. , Singh, M. F. , & Hamer, D. H. (2004). Vitamin E and respiratory tract infections in elderly nursing home residents: A randomized controlled trial. Jama‐Journal of the American Medical Association, 292(7), 828–836. 10.1001/jama.292.7.828 PMC2377357 15315997

[fft2119-bib-0140] Mhatre, S. , Srivastava, T. , Naik, S. , & Patravale, V. (2021). Antiviral activity of green tea and black tea polyphenols in prophylaxis and treatment of COVID‐19: A review. Phytomedicine, 85, 153286. 32741697 10.1016/j.phymed.2020.153286 PMC7367004

[fft2119-bib-0141] Mikkelsen, K. , & Apostolopoulos, V. (2019). Vitamin B1, B2, B3, B5, and B6 and the immune system nutrition and immunity (pp. 115–125). Springer.

[fft2119-bib-0142] Milewska, A. , Kaminski, K. , Ciejka, J. , Kosowicz, K. , Zeglen, S. , Wojarski, J. , Nowakowska, M. , Szczubiałka, K. , & Pyrc, K. (2016). HTCC: Broad range inhibitor of coronavirus entry. Plos One, 11(6), e0156552. 27249425 10.1371/journal.pone.0156552 PMC4889042

[fft2119-bib-0143] Miotto, M. , Di Rienzo, L. , Bò, L. , Boffi, A. , Ruocco, G. , & Milanetti, E. (2021). Molecular mechanisms behind anti SARS‐CoV‐2 action of lactoferrin. Frontiers in Molecular Biosciences, 8, 25. 10.3389/fmolb.2021.607443 PMC7917183 33659275

[fft2119-bib-0144] Mocchegiani, E. , & Muzzioli, M. (2000). Therapeutic application of zinc in human immunodeficiency virus against opportunistic infections. Journal of Nutrition, 130(5), 1424s–1431s. 10.1093/jn/130.5.1424S 10801955

[fft2119-bib-0145] Moghaddam, A. D. , Garavand, F. , Razavi, S. H. , & Talatappe, H. D. (2018). Production of saffron‐based probiotic beverage by lactic acid bacteria. Journal of Food Measurement and Characterization, 12(4), 2708–2717.

[fft2119-bib-0146] Mohammadi, N. , & Shaghaghi, N. (2020). Inhibitory effect of eight secondary metabolites from conventional medicinal plants on COVID_19 virus protease by molecular docking analysis. Biological and Medicinal Chemistry. 10.26434/chemrxiv.11987475.v1

[fft2119-bib-0147] Mohanty, D. P. , Mohapatra, S. , Misra, S. , & Sahu, P. S. (2016). Milk derived bioactive peptides and their impact on human health: A review. Saudi Journal of Biological Sciences, 23(5), 577–583. 10.1016/j.sjbs.2015.06.005 27579006 PMC4992109

[fft2119-bib-0149] Mozaffarieh, M. , Sacu, S. , & Wedrich, A. (2003). The role of the carotenoids, lutein and zeaxanthin, in protecting against age‐related macular degeneration: A review based on controversial evidence. Nutrition Journal, 2, 20. 10.1186/1475-2891-2-20 14670087 PMC305368

[fft2119-bib-0150] Muchtaridi, M. , Fauzi, M. , Ikram, N. K. K. , Gazzali, A. M. , & Wahab, H. A. (2020). Natural flavonoids as potential angiotensin‐converting enzyme 2 inhibitors for anti‐SARS‐CoV‐2. Molecules, 25(17), 3980. 10.3390/molecules25173980 PMC7504743 32882868

[fft2119-bib-0151] Mulangu, S. , Dodd, L. E. , Davey, R. T. , Tshiani Mbaya, O. , Proschan, M. , Mukadi, D. , Lusakibanza Manzo, M. , Nzolo, D. , Tshomba Oloma, A. , Ibanda, A. , Ali, R. , Coulibaly, S. , Levine, A. C. , Grais, R. , Diaz, J. , Lane, H. C. , Muyembe‐Tamfum, J.‐J. , & The PALM Writing Group . (2019). A randomized, controlled trial of Ebola virus disease therapeutics. New England Journal of Medicine, 381(24), 2293–2303. 10.1056/NEJMoa1910993 PMC10680050 31774950

[fft2119-bib-0152] Murphy, E. A. , Davis, J. M. , Brown, A. S. , Carmichael, M. D. , Carson, J. A. , Van Rooijen, N. , Ghaffar, A. , & Mayer, E. P. (2008). Benefits of oat β‐glucan on respiratory infection following exercise stress: Role of lung macrophages. American Journal of Physiology‐Regulatory, Integrative and Comparative Physiology, 294(5), R1593–R1599. 10.1152/ajpregu.00562.2007 18353878

[fft2119-bib-0284] Nie, L. , Wu, Y. , Dai, Z. , Ma, S. (2020). Antiviral activity of Isatidis Radix derived glucosinolate isomers and their breakdown products against influenza A in vitro/ovo and mechanism of action. Journal of Ethnopharmacology, 251, 112550. 10.1016/j.jep.2020.112550 31918015 PMC7126217

[fft2119-bib-0154] Nile, S. H. , & Park, S. W. (2014). Edible berries: Bioactive components and their effect on human health. Nutrition, 30(2), 134–144. 24012283 10.1016/j.nut.2013.04.007

[fft2119-bib-0155] Okai, Y. , & HigashiOkai, K. (1996). Possible immunomodulating activities of carotenoids in in vitro cell culture experiments. International Journal of Immunopharmacology, 18(12), 753–758. 10.1016/S0192-0561(97)85558-0 9172019

[fft2119-bib-0156] O'keefe, B. R. , Giomarelli, B. , Barnard, D. L. , Shenoy, S. R. , Chan, P. K. S. , Mcmahon, J. B. , Palmer, K. E. , Barnett, B. W. , Meyerholz, D. K. , Wohlford‐Lenane, C. L. , & Mccray, P. B. (2010). Broad‐spectrum in vitro activity and in vivo efficacy of the antiviral protein griffithsin against emerging viruses of the family coronaviridae. Journal of Virology, 84(5), 2511–2521. 10.1128/Jvi.02322-09 20032190 PMC2820936

[fft2119-bib-0157] Olivares, M. , Díaz‐Ropero, M. P. , Sierra, S. , Lara‐Villoslada, F. , Fonollá, J. , Navas, M. , Rodríguez, J. M. , & Xaus, J. (2007). Oral intake of Lactobacillus fermentum CECT5716 enhances the effects of influenza vaccination. Nutrition, 23(3), 254–260. 10.1016/j.nut.2007.01.004 17352961

[fft2119-bib-0158] Pandit, J. , Aqil, M. , & Sultana, Y. (2016). Nanoencapsulation technology to control release and enhance bioactivity of essential oils. Encapsulations, 2, 597–640. 10.1016/B978-0-12-804307-3.00014-4

[fft2119-bib-0159] Pang, R. , Tao, J.‐Y. , Zhang, S.‐L. , Zhao, L. , Yue, X. , Wang, Y.‐F. , Ye, P. , Dong, J.‐H. , Zhu, Y. , & Wu, J.‐G. (2010). In vitro antiviral activity of lutein against hepatitis B virus. Phytotherapy Research, 24(11), 1627–1630. 10.1002/ptr.3155 21031619

[fft2119-bib-0160] Paraiso, I. L. , Revel, J. S. , & Stevens, J. F. (2020). Potential use of polyphenols in the battle against COVID‐19. Current Opinion in Food Science, 32, 149–155. 32923374 10.1016/j.cofs.2020.08.004 PMC7480644

[fft2119-bib-0161] Peshev, D. , & Van den Ende, W. (2014). Fructans: Prebiotics and immunomodulators. Journal of Functional Foods, 8, 348–357. 10.1016/j.jff.2014.04.005

[fft2119-bib-0271] Pilau, M. R. , Alves, S. H. , Weiblen, R. , Arenhart, S. , Cueto, A. P. , Lovato, L. T. (2011). Antiviral activity of the Lippia graveolens (Mexican oregano) essential oil and its main compound carvacrol against human and animal viruses. Brazilian Journal of Microbiology, 42(4), 1616–1624. 10.1590/s1517-83822011000400049 24031796 PMC3768712

[fft2119-bib-0162] Polya, G. M. (2003). Protein and non‐protein protease inhibitors from plants. In Studies in natural products chemistry (Vol. 29, pp. 567–641). Elsevier.

[fft2119-bib-0163] Pyrć, K. , Milewska, A. , Duran, E. B. , Botwina, P. , Lopes, R. , ArenasPinto, A. et al. (2020) SARS‐CoV‐2 inhibition in human airway epithelial cells using a mucoadhesive, amphiphilic chitosan that may serve as an anti‐viral nasal spray. bioRxiv. 10.1101/2020.12.10.413609 PMC8501059 34625610

[fft2119-bib-0164] Qin, C. , Zhou, L. , Hu, Z. , Zhang, S. , Yang, S. , Tao, Y. , Xie, C. , Ma, K. , Shang, K. , Wang, W. , & Tian, D.‐S. (2020). Dysregulation of immune response in patients with coronavirus 2019 (COVID‐19) in Wuhan, China. Clinical Infectious Diseases, 71(15), 762–768. 32161940 10.1093/cid/ciaa248 PMC7108125

[fft2119-bib-0165] Raha, S. , Mallick, R. , Basak, S. , & Duttaroy, A. K. (2020). Is copper beneficial for COVID‐19 patients? Medical Hypotheses, 142, 109814. 10.1016/j.mehy.2020.109814 PMC7199671 32388476

[fft2119-bib-0166] Ramassamy, C. (2006). Emerging role of polyphenolic compounds in the treatment of neurodegenerative diseases: A review of their intracellular targets. European Journal of Pharmacology, 545(1), 51–64. 16904103 10.1016/j.ejphar.2006.06.025

[fft2119-bib-0167] Rao, A. R. , Sindhuja, H. N. , Dharmesh, S. M. , Sankar, K. U. , Sarada, R. , & Ravishankar, G. A. (2013). Effective inhibition of skin cancer, tyrosinase, and antioxidative properties by astaxanthin and astaxanthin esters from the green alga haematococcus pluvialis. Journal of Agricultural and Food Chemistry, 61(16), 3842–3851. 10.1021/jf304609j 23473626

[fft2119-bib-0168] Rashidinejad, A. , Birch, E. J. , & Everett, D. W. (2016). Interactions between milk fat globules and green tea catechins. Food Chemistry, 199, 347–355. 26775981 10.1016/j.foodchem.2015.12.030

[fft2119-bib-0169] Rashidinejad, A. , Birch, E. J. , Sun‐Waterhouse, D. , & Everett, D. W. (2017). Addition of milk to tea infusions: Helpful or harmful? Evidence from in vitro and in vivo studies on antioxidant properties. Critical Reviews in Food Science and Nutrition, 57(15), 3188–3196. 26517348 10.1080/10408398.2015.1099515

[fft2119-bib-0170] Read, S. A. , Obeid, S. , Ahlenstiel, C. , & Ahlenstiel, G. (2019). The role of zinc in antiviral immunity. Advances in Nutrition, 10(4), 696–710. 10.1093/advances/nmz013 31305906 PMC6628855

[fft2119-bib-0171] Real, E. , Rain, J.‐C. , Battaglia, V. , Jallet, C. , Perrin, P. , Tordo, N. , Chrisment, P. , D'alayer, J. , Legrain, P. , & Jacob, Y. (2004). Antiviral drug discovery strategy using combinatorial libraries of structurally constrained peptides. Journal of Virology, 78(14), 7410–7417. 10.1128/Jvi.78.14.7410-7417.2004 15220414 PMC434122

[fft2119-bib-0173] Regnault‐Roger, C. , Vincent, C. , & Arnason, J. T. (2012). Essential oils in insect control: Low‐risk products in a high‐stakes world. Annual Review of Entomology, 57, 405–424. 10.1146/annurev-ento-120710-100554 21942843

[fft2119-bib-0174] Rehman, A. , Ahmad, T. , Aadil, R. M. , Spotti, M. J. , Bakry, A. M. , Khan, I. M. , Zhao, L. , Riaz, T. , & Tong, Q. Y. (2019). Pectin polymers as wall materials for the nano‐encapsulation of bioactive compounds. Trends in Food Science & Technology, 90, 35–46. 10.1016/j.tifs.2019.05.015

[fft2119-bib-0175] Rehman, A. , Jafari, S. M. , Tong, Q. , Riaz, T. , Assadpour, E. , Aadil, R. M. , Niazi, S. , Khan, I. M. , Shehzad, Q. , Ali, A. , & Khan, S. (2020a). Drug nanodelivery systems based on natural polysaccharides against different diseases. Advances in Colloid and Interface Science, 284, 102251. 10.1016/j.cis.2020.102251 32949812

[fft2119-bib-0176] Rehman, A. , Tong, Q. , Jafari, S. M. , Assadpour, E. , Shehzad, Q. , Aadil, R. M. , Iqbal, M. W. , Rashed, M. M. A. , Mushtaq, B. S. , & Ashraf, W. (2020b). Carotenoid‐loaded nanocarriers: A comprehensive review. Advances in Colloid and Interface Science, 275, 102048. 10.1016/j.cis.2019.102048 31757387

[fft2119-bib-0177] Ren, G. M. , Xu, L. M. , Lu, T. Y. , & Yin, J. S. (2018). Structural characterization and antiviral activity of lentinan from Lentinus edodes mycelia against infectious hematopoietic necrosis virus. International Journal of Biological Macromolecules, 115, 1202–1210. 10.1016/j.ijbiomac.2018.04.132 29704603

[fft2119-bib-0178] Rerksuppaphol, S. , & Rerksuppaphol, L. (2019). A randomized controlled trial of zinc supplementation in the treatment of acute respiratory tract infection in Thai children. Pediatric Reports, 11(2), 15–20. 10.4081/pr.2019.7954 PMC6548996 31214301

[fft2119-bib-0179] Rothan, H. A. , & Byrareddy, S. N. (2020). The epidemiology and pathogenesis of coronavirus disease (COVID‐19) outbreak. Journal of Autoimmunity, 109, 102433. 32113704 10.1016/j.jaut.2020.102433 PMC7127067

[fft2119-bib-0180] Runfeng, L. , Yunlong, H. , Jicheng, H. , Weiqi, P. , Qinhai, M. , Yongxia, S. , Chufang, L. , Jin, Z. , Zhenhua, J. , Haiming, J. , Kui, Z. , Shuxiang, H. , Jun, D. , Xiaobo, L. , Xiaotao, H. , Lin, W. , Nanshan, Z. , & Zifeng, Y. (2020). Lianhuaqingwen exerts anti‐viral and anti‐inflammatory activity against novel coronavirus (SARS‐CoV‐2). Pharmacological Research, 156, 104761. 32205232 10.1016/j.phrs.2020.104761 PMC7102548

[fft2119-bib-0181] Saavedra, M. , Borges, A. , Dias, C. , Aires, A. , Bennett, R. , Rosa, E. , & Simões, M. (2010). Antimicrobial activity of phenolics and glucosinolate hydrolysis products and their synergy with streptomycin against pathogenic bacteria. Medicinal Chemistry (Shāriqah (United Arab Emirates)), 6, 174–183. 10.2174/1573406411006030174 20632977

[fft2119-bib-0182] Sabry, W. , Elemary, M. , Burnouf, T. , Seghatchian, J. , & Goubran, H. (2020). Vitamin B12 deficiency and metabolism‐mediated thrombotic microangiopathy (MM‐TMA). Transfusion and Apheresis Science, 59(1), 102717. 31902683 10.1016/j.transci.2019.102717

[fft2119-bib-0183] Saedisomeolia, A. , Wood, L. G. , Garg, M. L. , Gibson, P. G. , & Wark, P. A. B. (2009). Lycopene enrichment of cultured airway epithelial cells decreases the inflammation induced by rhinovirus infection and lipopolysaccharide. Journal of Nutritional Biochemistry, 20(8), 577–585. 10.1016/j.jnutbio.2008.06.001 18824341

[fft2119-bib-0184] Saladino, F. , Bordin, K. , Luciano, F. B. , Franzón, M. F. , Mañes, J. , & Meca, G. (2017). Antimicrobial activity of the glucosinolates. In J.‐M. Mérillon & K. G. Ramawat (Eds.), Glucosinolates (pp. 249–274). Springer International Publishing.

[fft2119-bib-0185] Salaris, C. , Scarpa, M. , Elli, M. , Bertolini, A. , Guglielmetti, S. , Pregliasco, F. , Blandizzi, C. , Brun, P. , & Castagliuolo, I. (2021). Protective effects of lactoferrin against SARS‐CoV‐2 infection in vitro. Nutrients, 13(2), 328. 33498631 10.3390/nu13020328 PMC7911668

[fft2119-bib-0186] Sánchez, A. , & Vázquez, A. (2017). Bioactive peptides: A review. Food Quality and Safety, 1(1), 29–46.

[fft2119-bib-0187] Sano, E. , Miyauchi, R. , Takakura, N. , Yamauchi, K. , Murata, E. , Le, Q. T. , & Katunuma, N. (2005). Cysteine protease inhibitors in various milk preparations and its importance as a food. Food Research International, 38(4), 427–433.

[fft2119-bib-0188] Sarlak, Z. , Rouhi, M. , Mohammadi, R. , Khaksar, R. , Mortazavian, A. M. , Sohrabvandi, S. , & Garavand, F. (2017). Probiotic biological strategies to decontaminate aflatoxin M1 in a traditional Iranian fermented milk drink (Doogh). Food Control, 71, 152–159.

[fft2119-bib-0189] Sarmadi, B. H. , & Ismail, A. (2010). Antioxidative peptides from food proteins: A review. Peptides, 31(10), 1949–1956. 10.1016/j.peptides.2010.06.020 20600423

[fft2119-bib-0190] Saura‐Calixto, F. (1998). Antioxidant dietary fiber product: A new concept and a potential food ingredient. Journal of Agricultural and Food Chemistry, 46(10), 4303–4306.

[fft2119-bib-0191] Saura‐Calixto, F. , Pérez‐Jiménez, J. , & Goni, I. (2010). Dietary fiber and associated antioxidants in fruit and vegetables. Fruit and Vegetable Phytochemicals, 223–234. 10.1002/9780813809397.ch8

[fft2119-bib-0192] Scalbert, A. , & Williamson, G. (2000). Dietary intake and bioavailability of polyphenols. The Journal of Nutrition, 130(8), 2073S–2085S. 10917926 10.1093/jn/130.8.2073S

[fft2119-bib-0193] Schnitzler, P. , Schuhmacher, A. , Astani, A. , & Reichling, J. (2008). Melissa officinalis oil affects infectivity of enveloped herpesviruses. Phytomedicine, 15(9), 734–740. 10.1016/j.phymed.2008.04.018 18693101

[fft2119-bib-0194] Seo, B. J. , Mun, M. R. , Kim, C.‐J. , Lee, I. , Kim, H. , & Park, Y.‐H. (2010). Putative probiotic Lactobacillus spp. from porcine gastrointestinal tract inhibit transmissible gastroenteritis coronavirus and enteric bacterial pathogens. Tropical Animal Health and Production, 42(8), 1855–1860. 20623187 10.1007/s11250-010-9648-5 PMC7089342

[fft2119-bib-0195] Serrano, G. , Kochergina, I. , Albors, A. , Diaz, E. , Oroval, M. , Hueso, G. , & Serrano, J. M. (2020). Liposomal lactoferrin as potential preventative and cure for COVID‐19. International Journal of Research in Health Sciences, 8(1), 8–15.

[fft2119-bib-0196] Sesso, H. D. , Liu, S. M. , Gaziano, J. M. , & Buring, J. E. (2003). Dietary lycopene, tomato‐based food products and cardiovascular disease in women. Journal of Nutrition, 133(7), 2336–2341. 10.1093/jn/133.7.2336 12840203

[fft2119-bib-0197] Shaghaghi, N. (2020). Molecular docking study of novel COVID‐19 protease with low risk terpenoides compounds of plants. ChemRxiv, 10.26434/chemrxiv.11935722.v1.

[fft2119-bib-0198] Shah, D. , Sachdev, H. S. , Gera, T. , De‐Regil, L. M. , & Pena‐Rosas, J. P. (2016). Fortification of staple foods with zinc for improving zinc status and other health outcomes in the general population. Cochrane Database of Systematic Reviews, (6), CD010697. 10.1002/14651858.CD010697.pub2 PMC8627255 27281654

[fft2119-bib-0199] Shahidi, F. , & Hossain, A. (2018). Bioactives in spices, and spice oleoresins: Phytochemicals and their beneficial effects in food preservation and health promotion. Journal of Food Bioactives, 3, 8–75.

[fft2119-bib-0200] Shahzad, F. , Anderson, D. , & Najafzadeh, M. (2020). The antiviral, anti‐inflammatory effects of natural medicinal herbs and mushrooms and SARS‐CoV‐2 infection. Nutrients, 12(9), 2573. 10.3390/nu12092573 PMC7551890 32854262

[fft2119-bib-0201] Shakoor, H. , Feehan, J. , Mikkelsen, K. , Al Dhaheri, A. S. , Ali, H. I. , Platat, C. , Ismail, L. C. , Stojanovska, L. , & Apostolopoulos, V. (2021). Be well: A potential role for vitamin B in COVID‐19. Maturitas, 144, 108–111. 32829981 10.1016/j.maturitas.2020.08.007 PMC7428453

[fft2119-bib-0202] Shereen, M. A. , Khan, S. , Kazmi, A. , Bashir, N. , & Siddique, R. (2020). COVID‐19 infection: Origin, transmission, and characteristics of human coronaviruses. Journal of Advanced Research, 24, 91–98. 32257431 10.1016/j.jare.2020.03.005 PMC7113610

[fft2119-bib-0203] Sheybani, Z. , Dokoohaki, M. H. , Negahdaripour, M. , Dehdashti, M. , Zolghadr, H. , Moghadami, M. , Masoompour, S. M. , & Zolghadr, A. R. (2020). The role of folic acid in the management of respiratory disease caused by COVID‐19. 10.26434/chemrxiv.12034980.v1

[fft2119-bib-0204] Shojadoost, B. , Kulkarni, R. R. , Yitbarek, A. , Laursen, A. , Taha‐Abdelaziz, K. , Negash Alkie, T. , Barjesteh, N. , Quinteiro‐Filho, W. M. , Smith, T. K. , & Sharif, S. (2019). Dietary selenium supplementation enhances antiviral immunity in chickens challenged with low pathogenic avian influenza virus subtype H9N2. Veterinary Immunology and Immunopathology, 207, 62–68. 10.1016/j.vetimm.2018.12.002 30593352

[fft2119-bib-0206] Singhal, T. (2020). A review of coronavirus disease‐2019 (COVID‐19). Indian Journal of Pediatrics, 87(4), 281–286. 10.1007/s12098-020-03263-6 32166607 PMC7090728

[fft2119-bib-0207] Sirichokchatchawan, W. , Temeeyasen, G. , Nilubol, D. , & Prapasarakul, N. (2018). Protective effects of cell‐free supernatant and live lactic acid bacteria isolated from Thai pigs against a pandemic strain of porcine epidemic diarrhea virus. Probiotics and Antimicrobial Proteins, 10(2), 383–390. 28434154 10.1007/s12602-017-9281-y PMC7091344

[fft2119-bib-0275] Song, S. , Peng, H. , Wang, Q. , Liu, Z. , Dong, X. , Wen, C. , Ai, C. , Zhang, Y. , Wang, Z. , Zhu, B. (2020). Inhibitory activities of marine sulfated polysaccharides against SARS‐CoV‐2. Food & Function, 11(9), 7415–7420. 10.1039/d0fo02017f 32966484

[fft2119-bib-0268] Snene, A. , El Mokni, R. , Jmii, H. , Jlassi, I. , Jaïdane, H. , Falconieri, D. , Piras, A. , Dhaouadi, H. , Porcedda, S. , Hammami, S. (2017). In vitro antimicrobial, antioxidant and antiviral activities of the essential oil and various extracts of wild (Daucus virgatus (Poir.) Maire) from Tunisia. Industrial Crops and Products, 109, 109–115. 10.1016/j.indcrop.2017.08.015

[fft2119-bib-0210] Steinmetz, K. A. , & Potter, J. D. (1996). Vegetables, fruit, and cancer prevention: A review. Journal of the American Dietetic Association, 96(10), 1027–1039. 8841165 10.1016/S0002-8223(96)00273-8

[fft2119-bib-0211] Suwannarach, N. , Kumla, J. , Sujarit, K. , Pattananandecha, T. , Saenjum, C. , & Lumyong, S. (2020). Natural bioactive compounds from fungi as potential candidates for protease inhibitors and immunomodulators to apply for Coronaviruses. Molecules, 25(8), 1800. 10.3390/molecules25081800 PMC7221821 32295300

[fft2119-bib-0212] Swamy, M. K. , Akhtar, M. S. , & Sinniah, U. R. (2016). Antimicrobial properties of plant essential oils against human pathogens and their mode of action: An updated review. Evidence‐Based Complementary and Alternative Medicine, 2016, 3012462. 28090211 10.1155/2016/3012462 PMC5206475

[fft2119-bib-0213] Tahaghoghi‐Hajghorbani, S. , Zafari, P. , Masoumi, E. , Rajabinejad, M. , Jafari‐Shakib, R. , Hasani, B. , & Rafiei, A. (2020). The role of dysregulated immune responses in COVID‐19 pathogenesis. Virus Research, 290, 198197. 33069815 10.1016/j.virusres.2020.198197 PMC7561578

[fft2119-bib-0214] Tamargo, J. , Le Heuzey, J.‐Y. , & Mabo, P. (2015). Narrow therapeutic index drugs: A clinical pharmacological consideration to flecainide. European Journal of Clinical Pharmacology, 71(5), 549–567. 25870032 10.1007/s00228-015-1832-0 PMC4412688

[fft2119-bib-0215] Tan, C. W. , Ho, L. P. , Kalimuddin, S. , Cherng, B. P. Z. , Teh, Y. E. , Thien, S. Y. , Wong, H. M. , Tern, P. J. W. , Chandran, M. , Chay, J. W. M. , Nagarajan, C. , Sultana, R. , Low, J. G. H. , & Ng, H. J. (2020a). A cohort study to evaluate the effect of combination vitamin D, magnesium and vitamin B12 (DMB) on progression to severe outcome in older COVID‐19 patients. medRxiv. 10.1101/2020.06.01.20112334 PMC7832811 33039952

[fft2119-bib-0216] Tan, C. W. , Ho, L. P. , Kalimuddin, S. , Cherng, B. P. Z. , Teh, Y. E. , Thien, S. Y. , Wong, H. M. , Tern, P. J. W. , Chandran, M. , Chay, J. W. M. , Nagarajan, C. , Sultana, R. , Low, J. G. H. , Ng, H. J. (2020b). Cohort study to evaluate the effect of vitamin D, magnesium, and vitamin B12 in combination on progression to severe outcomes in older patients with coronavirus (COVID‐19). Nutrition, 79‐80, 111017. 10.1016/j.nut.2020.111017 PMC7832811 33039952

[fft2119-bib-0217] Tariq, S. , Wani, S. , Rasool, W. , Shafi, K. , Bhat, M. A. , Prabhakar, A. , Shalla, A. H. , & Rather, M. A. (2019). A comprehensive review of the antibacterial, antifungal and antiviral potential of essential oils and their chemical constituents against drug‐resistant microbial pathogens. Microbial Pathogenesis, 134, 103580. 31195112 10.1016/j.micpath.2019.103580

[fft2119-bib-0218] Tarko, T. , Duda‐Chodak, A. , & Zajac, N. (2013). Digestion and absorption of phenolic compounds assessed by in vitro simulation methods. A review. Roczniki Państwowego Zakładu Higieny, 64(2), 79–84. 23987074

[fft2119-bib-0219] Tong, T. R. (2005). SARS‐CoV sampling from 3 portals. Emerging Infectious Diseases, 11(1), 167. 10.3201/eid1101.040645 PMC3294345 15714659

[fft2119-bib-0280] Tseliou, M. , Pirintsos, S. A. , Lionis, C. , Castanas, E. , Sourvinos, G. (2019). Antiviral effect of an essential oil combination derived from three aromatic plants (Coridothymus capitatus (L.) Rchb. f., Origanum dictamnus L. and Salvia fruticosa Mill.) against viruses causing infections of the upper respiratory tract. Journal of Herbal Medicine, 17‐18, 100288. 10.1016/j.hermed.2019.100288

[fft2119-bib-0220] Tsukahara, T. , Inatomi, T. , Otomaru, K. , Amatatsu, M. , Romero‐Pérez, G. A. , & Inoue, R. (2018). Probiotic supplementation improves reproductive performance of unvaccinated farmed sows infected with porcine epidemic diarrhea virus. Animal Science Journal, 89(8), 1144–1151. 29806133 10.1111/asj.13040 PMC7159621

[fft2119-bib-0221] Tutunchi, H. , Naeini, F. , Ostadrahimi, A. , & Hosseinzadeh‐Attar, M. J. (2020). Naringenin, a flavanone with antiviral and anti‐inflammatory effects: A promising treatment strategy against COVID‐19. Phytotherapy Research, 34(12), 3137–3147. 32613637 10.1002/ptr.6781 PMC7361426

[fft2119-bib-0223] Johns Hopkins University (2021). COVID‐19 Dashboard by the Center for Systems Science and Engineering at Johns Hopkins University. https://coronavirus.jhu.edu/map.html 10.1016/S1473-3099(22)00434-0 PMC9432867 36057267

[fft2119-bib-0224] Vafaeinezhad, A. , Atashzar, M. R. , & Baharlou, R. (2021). The immune responses against coronavirus infections: Friend or foe? International Archives of Allergy and Immunology, *182*(9), 1–14. 10.1159/000516038 PMC8247827 33951640

[fft2119-bib-0225] Velthuis, A. J. W. T. , van den Worm, S. H. E. , Sims, A. C. , Baric, R. S. , Snijder, E. J. , & van Hemert, M. J. (2010). Zn2+ inhibits Coronavirus and arterivirus RNA polymerase activity in vitro and zinc ionophores block the replication of these viruses in cell culture. PLoS Pathogens, 6(11), e1001176. 21079686 10.1371/journal.ppat.1001176 PMC2973827

[fft2119-bib-0226] Venkataraman, T. , & Frieman, M. B. (2017). The role of epidermal growth factor receptor (EGFR) signaling in SARS coronavirus‐induced pulmonary fibrosis. Antiviral Research, 143, 142–150. 10.1016/j.antiviral.2017.03.022 28390872 PMC5507769

[fft2119-bib-0227] Verhoeven, D. T. H. , Goldbohm, R. A. , vanPoppel, G. , Verhagen, H. , & vandenBrandt, P. A. (1996). Epidemiological studies on brassica vegetables and cancer risk. Cancer Epidemiology Biomarkers & Prevention, 5(9), 733–748. 8877066

[fft2119-bib-0228] Verma, S. (2020 ). In search of feasible interventions for the prevention and cure of novel coronavirus disease 2019. 10.31219/osf.io/q6tsc

[fft2119-bib-0229] Walsh, C. , Lane, J. A. , van Sinderen, D. , & Hickey, R. M. (2020). Human milk oligosaccharides: Shaping the infant gut microbiota and supporting health. Journal of Functional Foods, 72, 104074. 10.1016/j.jff.2020.104074 PMC7332462 32834834

[fft2119-bib-0232] Wang, M. , Phadke, M. , Packard, D. , Yadav, D. , & Gorelick, F. (2020). Zinc: Roles in pancreatic physiology and disease. Pancreatology, 20(7), 1413–1420. 10.1016/j.pan.2020.08.016 32917512 PMC7572834

[fft2119-bib-0235] Wang, X. , Wang, L. , Huang, X. , Ma, S. , Yu, M. , Shi, W. , Qiao, X. , Tang, L. , Xu, Y. , Li, Y. (2017). Oral Delivery of Probiotics Expressing Dendritic Cell‐Targeting Peptide Fused with Porcine Epidemic Diarrhea Virus COE Antigen: A Promising Vaccine Strategy against PEDV. Viruses, 9(11), 312. 10.3390/v9110312 PMC5707519 29068402

[fft2119-bib-0277] Wang, W. , Wu, J. , Zhang, X. , Hao, C. , Zhao, X. , Jiao, G. , Shan, X. , Tai, W. , Yu, G. (2017). Inhibition of Influenza A Virus Infection by Fucoidan Targeting Viral Neuraminidase and Cellular EGFR Pathway. Scientific Reports, 7(1), 10.1038/srep40760 PMC5240104 28094330

[fft2119-bib-0236] Wazir, S. M. , & Ghobrial, I. (2017). Copper deficiency, a new triad: Anemia, leucopenia, and myeloneuropathy. Journal of Community Hospital Internal Medicine Perspectives, 7(4), 265–268. 10.1080/20009666.2017.1351289 29046759 PMC5637704

[fft2119-bib-0237] Weizman, Z. , Asli, G. , & Alsheikh, A. (2005). Effect of a probiotic infant formula on infections in child care centers: Comparison of two probiotic agents. Pediatrics, 115(1), 5–9. 15629974 10.1542/peds.2004-1815

[fft2119-bib-0279] West, C. E. , Sijtsma, S. R. , Kouwenhoven, B. , Rombout, J. H. W. M. , van der Zijpp, A. J. (1992). Epithelia‐Damaging Virus Infections Affect Vitamin A Status in Chickens. The Journal of Nutrition, 122(2), 333–339. 10.1093/jn/122.2.333 1310111

[fft2119-bib-0281] Wen, C.‐C. , Kuo, Y.‐H. , Jan, J.‐T. , Liang, P.‐H. , Wang, S.‐Y. , Liu, H.‐G. , Lee, C.‐K. , Chang, S.‐T. , Kuo, C.‐J. , Lee, S.‐S. , Hou, C.‐C. , Hsiao, P.‐W. , Chien, S.‐C. , Shyur, L.‐F. , Yang, N.‐S. (2007). Specific Plant Terpenoids and Lignoids Possess Potent Antiviral Activities against Severe Acute Respiratory Syndrome Coronavirus. Journal of Medicinal Chemistry, 50(17), 4087–4095. 10.1021/jm070295s 17663539

[fft2119-bib-0238] Wessels, I. , & Rink, L. (2020). Micronutrients in autoimmune diseases: Possible therapeutic benefits of zinc and vitamin D. Journal of Nutritional Biochemistry, 77, 108240. 10.1016/j.jnutbio.2019.108240 31841960

[fft2119-bib-0239] Wessels, I. , Maywald, M. , & Rink, L. (2017). Zinc as a gatekeeper of immune function. Nutrients, 9(12), 1286. 10.3390/nu9121286 PMC5748737 29186856

[fft2119-bib-0240] Wichansawakun, S. , & Buttar, H. S. (2019). Antioxidant diets and functional foods promote healthy aging and longevity through diverse mechanisms of action: The role of functional food security in global health (pp. 541–563). Elsevier.

[fft2119-bib-0241] Williamson, G. , & Clifford, M. N. (2010). Colonic metabolites of berry polyphenols: The missing link to biological activity? British Journal of Nutrition, 104(S3), S48–S66. 10.1017/S0007114510003946 20955650

[fft2119-bib-0242] Wu, L. J. , Wang, W. , Zhang, X. S. , Zhao, X. , & Yu, G. L. (2016). Anti‐HBV activity and mechanism of marine‐derived polyguluronate sulfate (PGS) in vitro. Carbohydrate Polymers, 143, 139–148. 10.1016/j.carbpol.2016.01.065 27083353

[fft2119-bib-0244] Xie, Z. , Shi, Y. , Wang, Z. , Wang, R. , & Li, Y. (2011). Biotransformation of glucosinolates epiprogoitrin and progoitrin to (R)‐and (S)‐goitrin in Radix isatidis. Journal of Agricultural and Food Chemistry, 59(23), 12467–12472. 22023255 10.1021/jf203321u

[fft2119-bib-0245] Xu, H. Q. , He, L. W. , Chen, J. , Hou, X. B. , Fan, F. T. , Wu, H. Y. , Zhu, H., & Guo, Y. Q. (2019). Different types of effective fractions from Radix Isatidis revealed a multiple‐target synergy effect against respiratory syncytial virus through RIG‐I and MDA5 signaling pathways, a pilot study to testify the theory of superposition of traditional Chinese Medicine efficacy. Journal of Ethnopharmacology, 239, 111901. 10.1016/j.jep.2019.111901 31051218

[fft2119-bib-0246] Yadav, D. , Hertan, H. I. , Schweitzer, P. , Norkus, E. P. , & Pitchumoni, C. S. (2002). Serum and liver micronutrient antioxidants and serum oxidative stress in patients with chronic hepatitis C. American Journal of Gastroenterology, 97(10), 2634–2639. 10.1111/j.1572-0241.2002.06041.x 12385452

[fft2119-bib-0247] Yagi, T. , Asakawa, A. , Ueda, H. , Ikeda, S. , Miyawaki, S. , & Inui, A. (2013). The role of zinc in the treatment of taste disorders. Recent Patents on Food, Nutrition & Agriculture, 5(1), 44–51. 10.2174/2212798411305010007 23305423

[fft2119-bib-0270] Yan, Y. , Chang, L. , Wang, L. (2020). Laboratory testing of SARS‐CoV, MERS‐CoV, and SARS‐CoV‐2 (2019‐nCoV): Current status, challenges, and countermeasures. Reviews in Medical Virology, 30(3), 10.1002/rmv.2106 PMC7235496 32302058

[fft2119-bib-0248] Yang, X. , Dai, T. , Zhou, X. , Qian, H. , Guo, R. , Lei, L. , Zhang, X. , Zhang, D. , Shi, L. , Cheng, Y. , Hu, J. , Guo, Y. , & Zhang, B. (2020a). Analysis of adaptive immune cell populations and phenotypes in the patients infected by SARS‐CoV‐2. MedRxiv. 10.1101/2020.03.23.20040675

[fft2119-bib-0249] Yang, Y. , Islam, M. S. , Wang, J. , Li, Y. , & Chen, X. (2020b). Traditional Chinese medicine in the treatment of patients infected with 2019‐new coronavirus (SARS‐CoV‐2): A review and perspective. International Journal of Biological Sciences, 16(10), 1708. 32226288 10.7150/ijbs.45538 PMC7098036

[fft2119-bib-0250] Yin, J. C. , Li, G. X. , Li, J. , Yang, Q. , & Ren, X. F. (2011). In vitro and in vivo effects of Houttuynia cordata on infectious bronchitis virus. Avian Pathology, 40(5), 491–498. 10.1080/03079457.2011.605107 21848486

[fft2119-bib-0255] Zhang, L. , & Liu, Y. H. (2020). Potential interventions for novel coronavirus in China: A systematic review. Journal of Medical Virology, 92(5), 479–490. 10.1002/jmv.25707 32052466 PMC7166986

[fft2119-bib-0256] Zhang, R. J. , Wu, W. H. , Zhang, Z. P. , Lv, S. S. , Xing, B. S. , & McClements, D. J. (2019). Impact of food emulsions on the bioaccessibility of hydrophobic pesticide residues in co‐ingested natural products: Influence of emulsifier and dietary fiber type. Journal of Agricultural and Food Chemistry, 67(21), 6032–6040. 10.1021/acs.jafc.8b06930 31083996

[fft2119-bib-0257] Zhang, S. , Liu, M. , Li, H. , Jiang, L. , Luo, Y. , & Sun, Q. (2013). The in vitro anti‐virus effects and dose‐effect relationship of epigoitrin and fructopyrano‐(1 → 4)‐glucopyranose based on “deletion/increment” strategy. Chinese Journal of New Drugs, 22(9), 1083–1087.

[fft2119-bib-0258] Zhang, Y. Y. , Li, S. , Wang, X. H. , Zhang, L. N. , & Cheung, P. C. K. (2011). Advances in lentinan: Isolation, structure, chain conformation and bioactivities. Food Hydrocolloids, 25(2), 196–206. 10.1016/j.foodhyd.2010.02.001

[fft2119-bib-0276] Zhao, Y. , Ran, Z. , Jiang, Q. , Hu, N. , Yu, B. , Zhu, L. , Shen, L. , Zhang, S. , Chen, L. , Chen, H. , Jiang, J. , Chen, D. (2019). Vitamin D Alleviates Rotavirus Infection through a Microrna‐155‐5p Mediated Regulation of the TBK1/IRF3 Signaling Pathway In Vivo and In Vitro. International Journal of Molecular Sciences, 20(14), 3562. 10.3390/ijms20143562 PMC6678911 31330869

[fft2119-bib-0259] Zhao, S. , Lin, Q. , Ran, J. , Musa, S. S. , Yang, G. , Wang, W. , Lou, Y. , Gao, D. , Yang, L. , He, D. , & Wang, M. H. (2020). Preliminary estimation of the basic reproduction number of novel coronavirus (2019‐nCoV) in China, from 2019 to 2020: A data‐driven analysis in the early phase of the outbreak. International Journal of Infectious Diseases, 92, 214–217. 32007643 10.1016/j.ijid.2020.01.050 PMC7110798

[fft2119-bib-0260] Zhou, J. , Du, J. , Huang, L. T. , Wang, Y. C. , Shi, Y. M. , & Lin, H. L. (2018). Preventive effects of vitamin D on seasonal influenza A in infants: A multicenter, randomized, open, controlled clinical trial. Pediatric Infectious Disease Journal, 37(8), 749–754. 10.1097/Inf.0000000000001890 29315160

[fft2119-bib-0261] Zhou, L. , Xie, M. , Yang, F. , & Liu, J. (2020). Antioxidant activity of high purity blueberry anthocyanins and the effects on human intestinal microbiota. LWT, 117, 108621.

[fft2119-bib-0263] Zuo, L. , Li, J. , Xu, J. , Yang, J. , Zhang, D. , & Tong, Y. (2007). Studies on chemical constituents in root of Isatis indigotica. Zhongguo Zhong yao za zhi = Zhongguo zhongyao zazhi = China Journal of Chinese Materia Medica, 32(8), 688–691. 17608220

[fft2119-bib-0264] Zuo, T. , Zhang, F. , Lui, G. C. Y. , Yeoh, Y. K. , Li, A. Y. L. , Zhan, H. , Wan, Y. , Chung, A. C. K. , Cheung, C. P. , Chen, N. , Lai, C. K. C. , Chen, Z. , Tso, E. Y. K. , Fung, K. S. C. , Chan, V. , Ling, L. , Joynt, G. , Hui, D. S. C. , Chan, F. K. L. , … Ng, S. C. (2020). Alterations in gut microbiota of patients with COVID‐19 during time of hospitalization. Gastroenterology, 159(3), 944–955. 32442562 10.1053/j.gastro.2020.05.048 PMC7237927

